# Maternal Western diet mediates susceptibility of offspring to Crohn’s-like colitis by deoxycholate generation

**DOI:** 10.1186/s40168-023-01546-6

**Published:** 2023-05-02

**Authors:** Chongyang Huang, Huishi Tan, Mengyao Song, Ke Liu, Hongbin Liu, Jun Wang, Yanqiang Shi, Fengyi Hou, Qian Zhou, Ruo Huang, Binghai Shen, Xinlong Lin, Xiaoming Qin, Fachao Zhi

**Affiliations:** 1 Guangdong Provincial Key Laboratory of Gastroenterology, Institute of Gastroenterology of Guangdong Province, Department of Gastroenterology, Nanfang Hospital, Southern Medical University, Guangzhou, China; 2 Department of Gastroenterology, The Second Affiliated Hospital of Guangzhou University of Chinese Medicine, Guangzhou, China; 3 State Key Laboratory of Organ Failure Research, National Clinical Research Center of Kidney Disease, Division of Nephrology, Nanfang Hospital, Southern Medical University, Guangzhou, China; 4 Institute of Dermatology and Venereology, Dermatology Hospital, Southern Medical University, Guangzhou, China

**Keywords:** Maternal factors, Western diet, Gut microbiota, Deoxycholic acid, Gasdermin D, Intestinal inflammation

## Abstract

**Background:**

The Western dietary pattern, characterized by high consumption of fats and sugars, has been strongly associated with an increased risk of developing Crohn’s disease (CD). However, the potential impact of maternal obesity or prenatal exposure to a Western diet on offspring’s susceptibility to CD remains unclear. Herein, we investigated the effects and underlying mechanisms of a maternal high-fat/high-sugar Western-style diet (WD) on offspring’s susceptibility to 2,4,6-Trinitrobenzenesulfonic acid (TNBS)-induced Crohn’s-like colitis.

**Methods:**

Maternal dams were fed either a WD or a normal control diet (ND) for eight weeks prior to mating and continued throughout gestation and lactation. Post-weaning, the offspring were subjected to WD and ND to create four groups: ND-born offspring fed a normal diet (N–N) or Western diet (N-W), and WD-born offspring fed a normal (W–N) or Western diet (W-W). At eight weeks of age, they were administered TNBS to induce a CD model.

**Results:**

Our findings revealed that the W–N group exhibited more severe intestinal inflammation than the N–N group, as demonstrated by a lower survival rate, increased weight loss, and a shorter colon length. The W–N group displayed a significant increase in *Bacteroidetes*, which was accompanied by an accumulation of deoxycholic acid (DCA). Further experimentation confirmed an increased generation of DCA in mice colonized with gut microbes from the W–N group. Moreover, DCA administration aggravated TNBS-induced colitis by promoting Gasdermin D (GSDMD)-mediated pyroptosis and IL-1beta (IL-1β) production in macrophages. Importantly, the deletion of GSDMD effectively restrains the effect of DCA on TNBS-induced colitis.

**Conclusions:**

Our study demonstrates that a maternal Western-style diet can alter gut microbiota composition and bile acid metabolism in mouse offspring, leading to an increased susceptibility to CD-like colitis. These findings highlight the importance of understanding the long-term consequences of maternal diet on offspring health and may have implications for the prevention and management of Crohn’s disease.

Video Abstract

**Supplementary Information:**

The online version contains supplementary material available at 10.1186/s40168-023-01546-6.

## Background

Crohn’s disease (CD) is a chronic inflammatory gastrointestinal disorder characterized by high infiltration of immune cells into the inflamed mucosa and excessive production of pro-inflammatory cytokines [1, 2]. CD is often diagnosed at a young age, and its incidence has been rising worldwide. Moreover, the incidence of pediatric CD globally increased from 5.2 to 7.9 per 100,000 children in the past few decades, potentially due to the current shift towards Westernized dietary habits [3–6].

Traditional Western dietary patterns are characterized by a high intake of dietary fat and sugar, with a lower intake of dietary fiber [7, 8], and long-term consumption of Western diets has been linked to metabolic problems and obesity [9]. A recent population-based cohort study found that exposure to a Western diet is correlated with an increased incidence of developing CD [10, 11].

A relatively stable gut microbial community is established early in life and can be influenced by various factors, including prenatal environment, mode of birth, and post-natal circumstances, which may alter the disease risk profile later in life [12–15]. Maternal obesity or maternally consumed unhealthy diets may predispose children to various metabolic disorders [16, 17]. Previous research demonstrated that infants born to obese mothers have significantly altered gut microbiota, potentially leading to increased inflammation and susceptibility to nonalcoholic fatty liver disease (NAFLD) [18]. Additionally, an animal study reported that a high-fat maternal diet affects gut microbiota composition and disrupts the intestinal barrier in their offspring, thereby increasing susceptibility to DSS-induced colitis in adulthood [19].

Bile acids (BAs) are cholesterol metabolites synthesized and conjugated by the liver [20]. BAs are transformed by the gut microbiota for the effective recycling of BAs in the body, and it is suggested that BAs not only rely on the gut microbiota for their metabolism but also have a direct impact on the composition and function of the intestinal microbiota [21–24]. Primary BAs are predominantly converted to secondary BAs, such as deoxycholic acid (DCA) and lithocholic acid (LCA), in the gut, where they act as signaling molecules binding to farnesoid X receptors (FXR) or G protein-coupled bile acid receptors (TGR5) [25]. However, an excess of BAs induced by a Western diet may disrupt intestinal homeostasis or potentially trigger inflammation [26, 27].

The relationship between a maternal Western diet (MWD) and increased CD incidence in their offspring remains unclear. In this study, we investigated the impact of a maternal high-fat/high-sugar Western diet on the composition of intestinal microbiota and metabolites in offspring, as well as their susceptibility to 2,4,6-Trinitrobenzenesulfonic acid (TNBS)-induced colitis. We observed a substantial increase in *Bacteroidetes* and fecal DCA levels in pups born and fostered by WD-dams. Notably, exogenous DCA effectively exacerbated experimentally induced CD. DCA also promoted Gasdermin D (GSDMD)-mediated pyroptosis and interleukin-1 beta (IL-1β) production in macrophages. These findings suggest a possible link between maternal Western diet exposure and increased CD susceptibility in offspring.

## Methods

### Antibodies and reagents

Anti-ZO-1 (GB111981), anti-Occludin (GB111401), anti-F4/80 (GB11027), anti-IL-1β (GB11113), anti-MUC2 (GB11344), Oil Red staining solution (G1015), and PAS staining solution (G1008) were purchased from Servicebio. Anti-GSDMD (ab209845) was purchased from Abcam.

High-fat purified rodent diet (HF60-112,252) and control diet (AIN93G-110700) were obtained from Dyet. D-glucose (118,327,010) was procured from Guanghua Technology. Lipopolysaccharides (297–473-0), vancomycin (1404–93-9), neomycin sulfate (1405–10-3), metronidazole (443–48-1), ampicillin (69–52-3), acetone (67–64-1), DCA (D2510) and picryl sulfonic acid solution TNBS (P2297) were obtained from Sigma-Aldrich. Olive oil (O815211) was obtained from MACKLIN. Recombinant mouse GM-CSF (96–315-03–20) was procured from Peprotech. ATP solution (C0550) was purchased from Solarbio. L-012 (120–04,891) was acquired from Wako Science. Mouse DCA ELISA Kit (MM-44754M1) was bought from MEIMIAN-bio. Cholestyramine resin (11,041–12-6) was obtained from MedChem Express.

### Mouse models

*GSDMD-KO* mice were kindly provided by Professor Feng Shao (National Institute of Biological Sciences, Beijing, China). Wild-type (WT) C57BL/6 J mice were purchased from Cyagen Biosciences and maintained under specific pathogen-free (SPF) conditions in accredited animal facilities at Southern Medical University. All animal research protocols were approved by the Southern Medical University Institutional Animal Care and Use Committee.

Female WT mice were fed either a high-fat/high-sugar (60% high-fat diet supplemented with 12% (w/vol) glucose water, HF/HS) or a control diet for 8 weeks. Afterward, female mice were mated with strain and age-matched WT males, then fed a control diet to produce offspring. The HF/HS diet mimicked a typical Western-style diet by combining high-fat food with a sugar-sweetened beverage. This dietary combination was chosen because the high-fat and sugar intake has been associated with a greater likelihood of CD [11]. Detailed dietary compositions and caloric contents of the two experimental diets are described in Additional file 1: Table S1 and Table S2. Female mice were randomly assigned to dietary interventions, ensuring that body weight was similar between the two groups at study initiation. After 8 weeks of dietary exposure, the metabolic status of female mice was recorded.

After becoming pregnant, the mother was exposed to either a normal diet (ND) or a western diet (WD) throughout the gestation and lactation period. At 4 weeks of age, the offspring mice were weaned and assigned to one of the following four groups: ND-born offspring were fed either an ND (N–N) or a WD (N-W), while WD-born offspring were fed either an ND (W–N) or a WD (W-W). A mouse model of TNBS-induced colitis was established as previously described [28]. Briefly, TNBS pre-sensitization solution was prepared by mixing 4 volumes of an acetone/olive oil solution (4:1) with 1 volume of 5% TNBS. TNBS solution was prepared by mixing 1 volume of 5% TNBS solution with 1 volume of absolute ethanol. Eight- to 10-week-old male mice were treated with TNBS pre-sensitization solution on the shaved skin. One week later, the mice were starved overnight and then intrarectally administered with TNBS solution. Throughout the experiment, the body weight and survival rate of the mice were monitored daily.

To remove intestinal bile acid, the mice were orally administered a single dose of cholestyramine resin (2.5 g/kg) per day for 5 days prior to TNBS treatment.

For exogenous DCA intervention, the mice were administered water containing 0.2% DCA for 4 weeks, followed by rectal administration of TNBS to induce colitis.

To remove intestinal microbiota, W–N and N–N mice were intragastrically administered antibiotics, including 100 mg/kg vancomycin, 200 mg/kg neomycin sulfate, 200 mg/kg metronidazole, and 200 mg/kg ampicillin, once a day for 3 days prior to TNBS treatment.

### Fecal microbiota transplantation (FMT)

The experiment on fecal bacteria transplantation was conducted with minor modifications as previously described [29]. Briefly, wild-type male C57BL/6 mice were administered intragastrically with antibiotic cocktails comprising 100 mg/kg vancomycin, 200 mg/kg neomycin sulfate, 200 mg/kg metronidazole and 200 mg/kg ampicillin for 3 days. Feces were collected from donor mice (W–N and N–N groups), resuspended in PBS at a concentration of 0.125 g/mL, and then orally administered to the mice by oral gavage of 0.2 mL once a day for 5 days. Then, they were subjected to colitis induction using TNBS.

### Imaging of intestinal inflammation in vivo

On day 4 following the TNBS treatment, the mice were anesthetized and intraperitoneally injected with L-012 (25 mg/kg). After 1 min, they were exposed to a Spectral Instruments In vivo Imaging system and images were captured and assessed in the designated group of mice.

### Human samples

Endoscopic colonic mucosal biopsy specimens were obtained from healthy donors and IBD patients who attended the Department of Gastroenterology at Nanfang Hospital. Diagnosis and clinical evaluations of disease activity were based on a standard combination of medical, endoscopic, histological, and radiologic standards. All intestinal pinch biopsies were obtained from consenting participants during normal endoscopies, following the guidelines of the Ethics Committee of Nanfang Hospital, Southern Medical University. Demographic characteristics are presented in Additional file 1: Table S3.

### Histochemistry and immunostaining

Colonic samples obtained from humans and mice were fixed in 4% paraformaldehyde for 24 h, embedded in paraffin, and 5-μm-thick sections were used for hematoxylin–eosin (H&E) or immunostaining. For immunohistochemistry and immunofluorescence staining, deparaffinized sections were subjected to quenching of endogenous peroxidase activity, antigen-retrieval, and subsequent blocking procedures. The slices were then incubated with primary antibodies at 4 °C overnight, followed by incubation with biotinylated secondary antibodies for 2 h at room temperature. Protein expression levels were analyzed and scored by experienced pathologists using Image-Pro Plus software 6.0.0.260 (Media Cybernetics Inc., Bethesda, MD).

### Isolation and culture of bone marrow-derived macrophages

Bone marrow-derived macrophages (BMDMs) were generated from bone marrow cells (BM cells) obtained from the thighbone and shinbone of 8- to 10-week-old male WT mice. The BM cells were cultured in Dulbecco’s modified Eagle’s medium (DMEM) at 37 °C and 5% CO2. To induce differentiation to BMDMs, the media were supplemented with 20% FBS, 100 U/mL penicillin, 100 µg/mL streptomycin, and 30 ng/mL GM-CSF for 7 days. New media were added every 2 days, and non-adherent cells were removed by washing with PBS. The adherent cells were harvested after 7 days, of which more than 95% were F4/80 + macrophages.

To induce pyroptotic cell death, BMDMs were stimulated with 100 ng/mL LPS for 4 h, followed by 5 mM ATP for 30 min. In the LPS + ATP plus DCA group, BMDMs were treated with 100 ng/mL LPS and 50 μM DCA for 4 h, and then stimulated with 5 mM ATP for 30 min.

### ELISA

DCA levels in the stools of mice were assessed using specific enzyme-linked immunosorbent assay (ELISA) kits (MM-44754M1; mmbio, Jiangsu Meimian industrial Co. Ltd., China) following the manufacturer’s instructions. A total of 100 mg stool was mashed with 1 mL phosphate buffer saline, and the stool supernatant was obtained by centrifugation at 300 × *g* for 5 min. The fecal supernatant was then diluted 10 times using the dilution buffer in the kit for sample preparation. A volume of 50 μL of the prepared samples and standards was added gradually to each well, followed by 100 μL of HRP-conjugate reagent. The plate was covered with an adhesive strip and incubated for 60 min at 37 °C. The procedure was repeated four times for each well, and the plate was washed five times. Chromogen Solution A (50 μL) and Chromogen Solution B (50 μL) were then added to each well, and the plate was incubated at 37 °C for 15 min in the dark. After that, 50 μL of Stop Solution was added to each well, and the color of the wells changed from blue to yellow. Optical density (OD) was evaluated at 450 nm using a microtiter plate reader within 10 min. The linear regression curve of the standards was plotted, and the concentration values of each sample were calculated according to the equation of the curve.

### Western blot

Western blot analysis was performed as previously described [30]. GAPDH was used as an endogenous control. Anti-GSDMD, anti-Caspase1, and anti-IL-1β were diluted to a ratio of 1:1000, while anti-GAPDH was diluted to a ratio of 1:2000. The secondary antibodies were diluted to a ratio of 1:4000. The density of the protein bands was quantified and analyzed using Alpha-View software 3.4.0.0 (Bio-Techne Corporation, Minneapolis, MN).

### Real-time PCR

Real-time PCR was performed as previously described [30]. Briefly, the mRNA levels of the target genes were normalized to that of 18S. For microbiota detection, the mRNA levels were normalized to that of 16S. The primers used for the target genes are shown in Additional file 1: Table S4.

### Intestinal microbiota analysis

The 16S rDNA gene sequencing procedure was performed by BGI Tech (Guangzhou, China). The PCR reaction system was configured using 30 ng of qualified genomic DNA samples and the corresponding fusion primers (338F: ACTCCTACGGGAGGCAGCAG, 806R: GGACTACHVGGGTWTCTAAT), and the corresponding PCR reaction parameters were set for PCR amplification. The PCR amplification products were purified, and library construction was completed. The libraries were assessed for fragment range and concentration using an Agilent 2100 Bioanalyzer. The libraries that passed the test were sequenced by selecting the appropriate platform (HiSeq/MiSeq) according to the insert size. The downstream data were filtered to remove low-quality reads, and the remaining high-quality clean data were used for post-analysis. The reads were stitched into tags based on the overlapping relationship between reads. The tags were then clustered into operational taxonomic units (OTUs) at a given similarity, and the OTUs were compared with the database for species annotation. Species annotation was performed based on the OTUs and the species annotation results for sample species complexity analysis and species difference analysis between groups.

### Untargeted metabolomics analysis process

The assessments of untargeted metabolomics were conducted by BGI Tech (Guangzhou, China). The experimental procedure included metabolite extraction and LC–MS/MS detection. Briefly, 25 mg tissues were weighed and extracted by directly adding 800 µL of precooled extraction reagent (methanol: acetonitrile: water (2:2:1, v/v/v)), internal standards mix was added for quality control of sample preparation. The samples were homogenized for 5 min using TissueLyser (JXFSTPRP, China), followed by 10 min of sonication and an hour of incubation at – 20 °C. The supernatant from the centrifugation of the samples was then transferred for vacuum freeze drying after 15 min at 25,000 rpm and 4 °C. The metabolites were resuspended in 600 µL of 10% methanol and sonicated for 10 min at 4 °C. After centrifuging for 15 min at 25,000 rpm, the supernatants were transferred to autosampler vials for LC–MS analysis. To evaluate the consistency of the entire LC–MS analysis, a quality control (QC) sample was created by pooling the same volume of each sample. We employed a tandem Q Exactive high-resolution mass spectrometer from Thermo Fisher Scientific (USA) and Waters 2D UPLC (USA) for metabolite separation and identification.

### Bile-acid detection and analysis

BA detection was performed by BGI Tech (Guangzhou, China). Briefly, the kit’s standard material CAL8 was utilized for gradient dilution to create the eight standard curve points CAL1-CAL8. Then, 25 mg of the sample was weighed and mixed with 500 μL of precooled ACN (5% NH4), shaken, and mixed for 10 min, then centrifuged at 25,000 × *g*, 4 °C for 15 min. Next, 400 μL of the supernatant was taken and dried with nitrogen, followed by reconstitution with 50 μL of 50% methanol and vortexing for 3 min. Sample, standard material, Blank (pure water), and QC (mixed with commercial serum) were each added to 50 μL of the reconstituted mixture, followed by the addition of 200 μL of DB (50% methanol) separately. To each, 150 μL of precipitant with internal standard was added, shaken for 2 min, and centrifuged at 4000 rpm and 4 °C for 30 min. The supernatant (80 μL) was taken and mixed with 80 μL of pure water, shaken for 2 min, and centrifuged (4000 rpm, 4 °C, 30 min) before undergoing LC–MS/MS analysis. The analytical device used in this experiment was a liquid-mass spectrometry system comprising QTS triple quadrupole mass spectrometry (Waters, USA) and ACQUITY UPLC I-Class ultra-high performance liquid chromatography (Waters, USA). In the MassLynx program, each MRM transition (ion pair) was automatically identified and integrated using the default parameters and manually examined. The bile acid content (A × B) was calculated, where A is the concentration value determined by integrating the peak area of the target index in the sample, and B is the sample dilution factor (default value, 1), with units of μg/mg.

### Correlation analysis of differential metabolites and microbial groups

The obtained omics data was subjected to preprocessing, including identification and quantification of metabolites and calculation of relative microbial abundance based on absolute abundance. Correlation analysis was then performed between metabolite intensity or module eigenvalues and microbial relative abundance using two techniques: Spearman rank correlation analysis and canonical correlation analysis (CCA). CCA was implemented using the mixOmics R package to link co-expression clusters of metabolites with microbial groups based on eigenvector values and relative microbial abundance. A positive correlation indicates a similar variation trend between a differential metabolite and a microbial group, while a negative correlation indicates an opposite variation trend.

### Statistical analysis

Statistical analyses were performed using GraphPad Prism software version 8.0.2.263 (GraphPad Software Inc., San Diego, CA) unless otherwise indicated. Further, unless otherwise stated, data are presented as mean ± SEM. Two-way ANOVA with the Tukey test was used to determine statistical significance for data with a 2 × 2 factorial design, while a two-tailed Student’s *t* test was used for all other analyses. *P* values < 0.05 were considered statistically significant.

## Results

### Maternal western diet aggravates TNBS-induced colitis in offspring

Female mice were fed either a Western-style diet (WD) or a Normal diet (ND) for 8 weeks before mating with males in a mixed cage. At the end of the 8th week, the metabolic status of the female mice was evaluated. The results showed that the female mice administered the WD gained more body weight, exhibited impaired glucose tolerance, and had higher parametrial white adipose tissue (WAT) accumulation than those given the ND (Figure S1a − e). Histological examination with oil red staining showed that mice exposed to WD had greater lipid accumulation in their liver (Figure S1f–h).

**Fig. 1:**
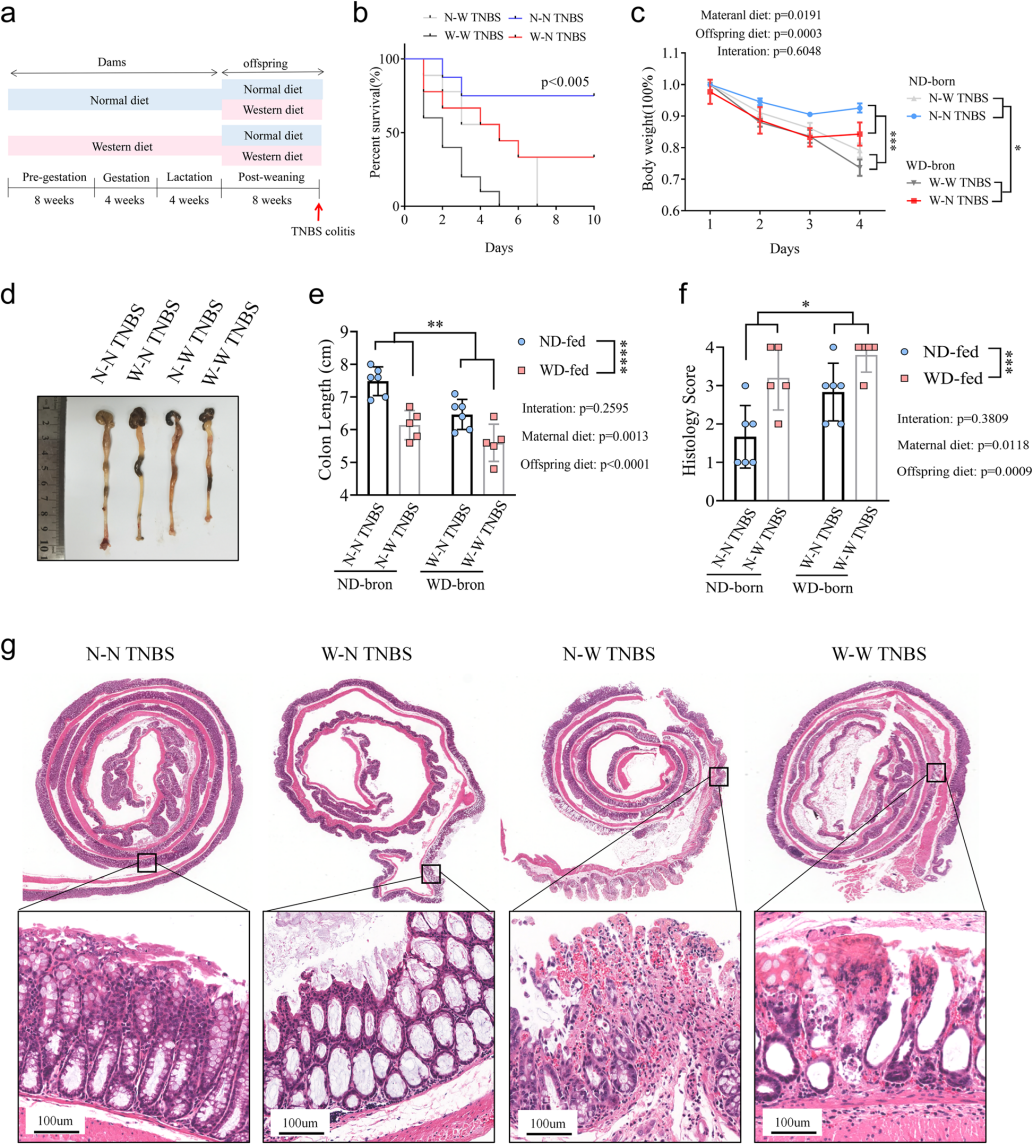
MWD aggravates TNBS-induced colitis in offspring. **a**–**g** Maternal dams were fed either a WD or ND for 8 weeks before mating and continued through gestation and lactation. Post-weaning pups were subjected to WD and ND to generate four groups: N-N, N-W, W-N, and W-W groups. At the age of 8 weeks, four groups of pups were treated with TNBS to induce a CD-like colitis model. **b** Survival rates of mice (*n* = 10) were monitored daily after TNBS administration. **c** Body weight changes of mice (*n* = 6) were monitored daily after TNBS administration. **d** Representative images of mouse colon in N-N, W-N, N-W, and W-W groups treated with TNBS. **e** The mice were sacrificed on day 4, and their colon lengths were measured. **f** and **g** Colon sections were examined histologically. **f** Histology scores for colonic inflammation were measured. **g** Representative images of the H&E-stained colon sections of indicated groups (scale bars 100 μm). **b**, **c**, **e**, and **f** Data represent means ± SEM; *P < 0.05; **P< 0.01; ***P < 0.001; ****P < 0.0001; by two-way ANOVA. The data shown are representative of three independent experiments

To elicit CD-like colitis, the offspring mice exposed to ND or WD were given TNBS at the age of 8 weeks (Fig. 1a). Our findings revealed that post-weaning mice given WD (N-W and W-W groups) had more severe colonic inflammation after being treated with TNBS than mice fed on ND (N–N and W–N groups) (Fig. 1b, c). Moreover, mice in the W–N group had more TNBS-induced intestinal inflammation than those in the N–N group, as demonstrated by the observed lower survival, shorter colon length, and higher histopathological scores (Fig. 1b–g). Therefore, the results suggest that both pre-weaning and post-weaning exposure to WD can impact offspring’s susceptibility to TNBS-induced colitis.

### Gut microbiota participates in the MWD-exacerbated intestinal inflammation

Considering that the intestinal barrier is a crucial determinant of susceptibility to intestinal inflammation, which is vulnerable to environmental factors such as dietary habits [3], we initially investigated the intestinal barrier integrity of offspring mice. Our data showed no significant differences in intestinal tight junctions between progeny mice that received different treatments (Figure S2a–e). However, post-weaning exposure to a WD significantly compromised the intestinal mucus barrier, while maternal factors associated with WD had little effect on the intestinal mucus barrier of offspring mice. These findings suggest that other factors may contribute to the exacerbation of intestinal inflammation (Figure S3a–d). Overall, post-weaning consumption of a WD may predominantly impact the mucus barrier and worsen intestinal inflammation, whereas the role of maternal factors remains unclear.

**Fig. 2:**
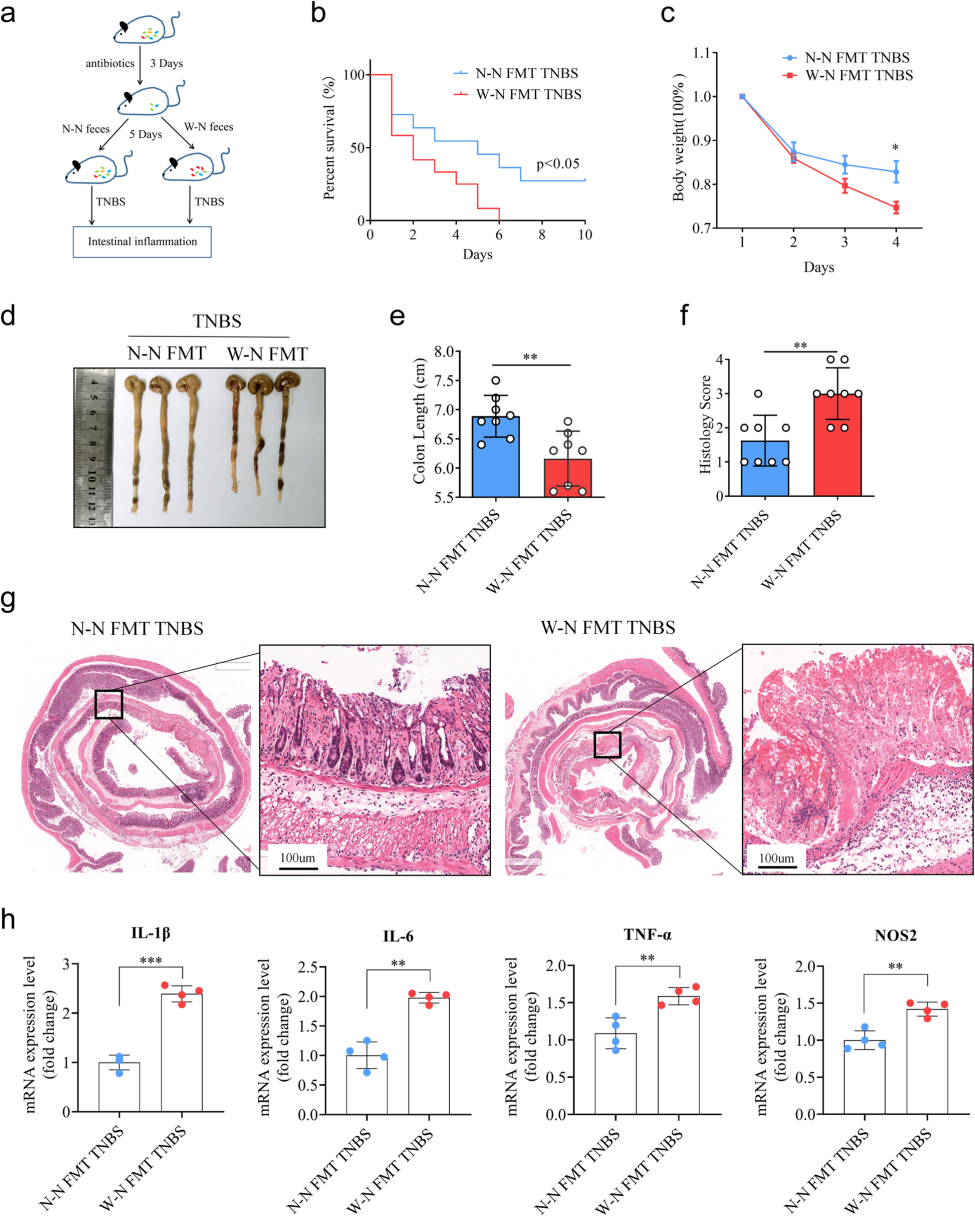
Altered gut microbiota associated with MWD exacerbates TNBS-induced colitis. **a**–**g** Littermate WT mice were administered antibiotics for 3 days to remove gut microbiota, and then colonized with gut microbiota from W-N and N-N groups for 5 days. After that, mice were rectally injected with TNBS to induce colitis. **b** Survival rates of mice (*n* = 10) were monitored daily after TNBS administration. **c** Body weight changes of mice (*n* = 8) were monitored daily after TNBS administration. **d** Representative images of mouse colon in the N-N FMT and W-N FMT groups treated with TNBS. **e** The mice were sacrificed on day 4, and colon lengths were measured. **f** and **g** Colon sections were examined histologically. **f** Histology scores for colonic inflammation were measured. **g** Representative images of the H&E-stained colon sections of indicated groups (scale bars 100 μm). **h** Relative mRNA levels of pro-inflammatory cytokines IL-1β, IL-6, TNF-α, and NOS2 were determined by real-time PCR and normalized to 18s. **b**, **c**, **e**, **f**, and **h** Data represent means ± SEM; *P < 0.05; **P < 0.01; ***P < 0.001; by unpaired Student’s *t* test. The data shown are representative of three independent experiments

Previous research has suggested that maternal factors can influence the gut microbiota in offspring mice [31]. Therefore, we hypothesized that the maternal effect on susceptibility to TNBS-induced colitis is related to altered microbiota in the offspring. To assess the connection between microbiota and phenotype, we used an antibiotic cocktail to eliminate the gut microbiota of mice in the W–N and N–N groups (Figure S4a). Remarkably, we found that the maternal effect on offspring susceptibility to colitis was significantly reduced upon removal of the gut microbiota, suggesting an indispensable role of the microbiota in the maternal effect (Figure S4b-f). We further conducted fecal microbiota transplantation (FMT) to investigate the role of MWD-associated gut microbiota (Fig. 2a). Consistently, mice receiving W–N gut microbiota transplantation (W–N FMT) had more severe TNBS-induced colitis than those receiving N–N gut microbiota transplantation (N–N FMT), as evidenced by the observed lower survival, greater weight loss, and shorter colon length (Fig. 2b–e). Moreover, histological analysis of colon sections from the W–N FMT group revealed more extensive damage to the intestinal epithelium and a greater number of immune cell infiltrations after TNBS treatment than the N–N FMT group (Fig. 2f–g). The W–N FMT group also exhibited increased gene expression of pro-inflammatory cytokines, including IL-6, IL-1β, TNF-α, and NOS2 (Fig. 2h). These analyses indicate that the MWD-derived gut microbiota could exacerbate TNBS-induced intestinal inflammation.

### MWD induces gut microbiota and bile acid metabolism shifts in the offspring

**Fig. 3:**
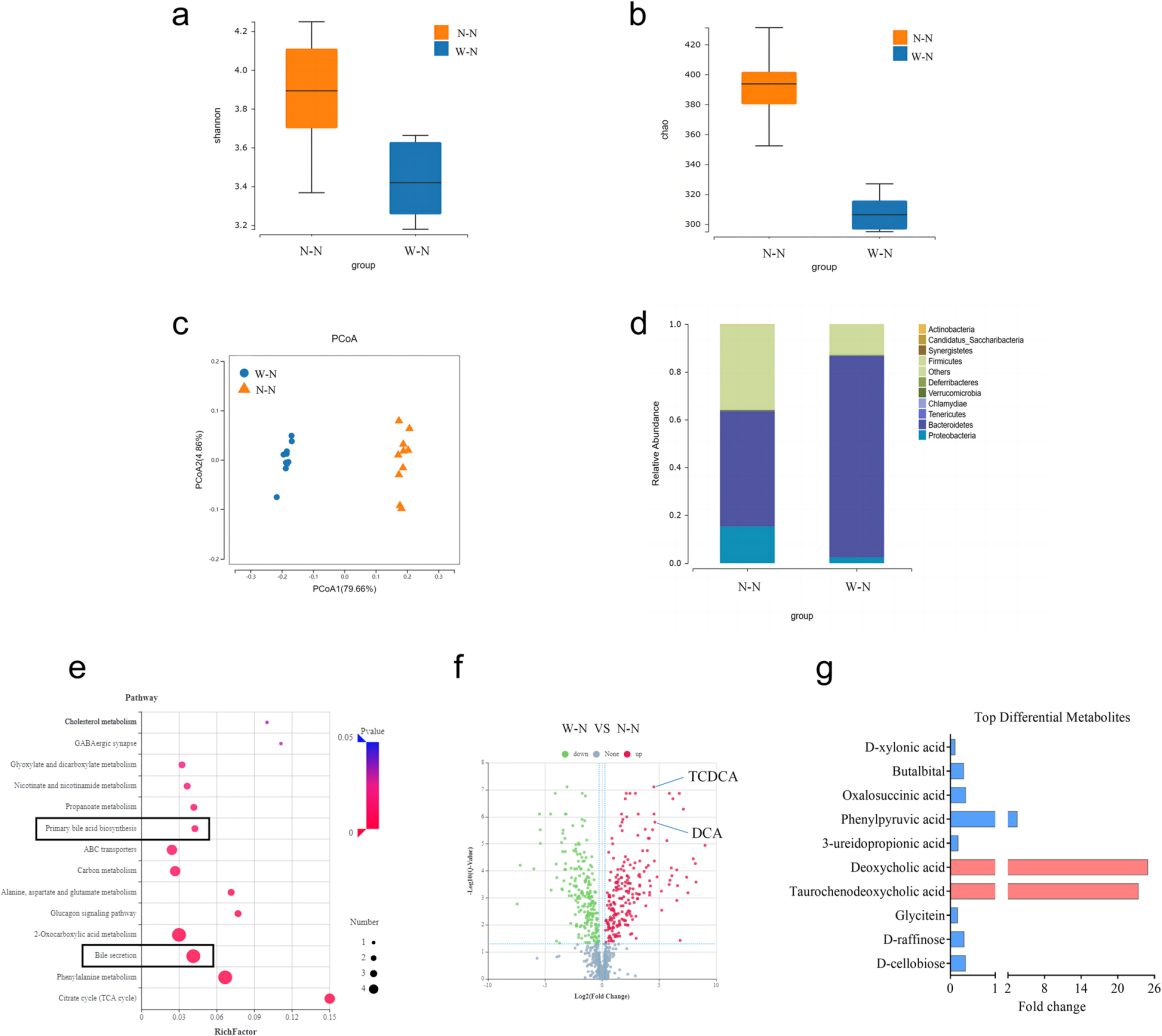
MWD induces intestinal microbiota and metabolite shifts in the offspring. Feces from the W-N and N-N group mice were collected for 16s rDNA analysis and untargeted metabolomics analysis. **a**, **b** α-diversity analysis of gut microbes reflected by Shannon and Chao indices. **c** Scatter plots of weighted PCoA for the microbial composition. **d** Relative abundance of gut microbiota at the phylum level. **e** KEGG pathway enrichment analyses of the upregulated differential metabolites in the W-N group. The dot size represents the number of differential metabolites, and the dot color represents the corresponding *p* value. **f** Scatter plot showing differential metabolites in the W-N group versus the N-N group. Differential metabolites were plotted based on their expression levels (log10 intensity). Red and green dots represented up- and downregulated genes, respectively. Differential bile acids were indicated. **g** Top 10 differential metabolites in the W-N group versus the N-N group. The *y* axis shows the name of metabolites, and the x-axis shows the fold change of metabolites

Further investigation into the altered gut microbiota, which contributes to colitis development due to MWD induction, was conducted in the W–N and N–N groups via 16S sequencing. The alpha diversity of the intestinal microbiota was found to be lower in the W–N group compared to the N–N group based on Shannon and chao studies (Fig. 3a, b). Additionally, PCoA analysis indicated significant variations in the communities of intestinal microbiota between the two groups (Fig. 3c). Analysis of the gut microbiota by phylum revealed that *Bacteroidetes* were more prevalent, while *Proteobacteria* and *Firmicutes* were less abundant in the W–N group compared to the N–N group (Fig. 3d and Figure S5a). BugBase analysis predicted increased pathogenicity and decreased stress resistance in the microbiota of mice in the W–N group compared to the N–N group (Figure S5b). Furthermore, Tax4Fun functional prediction of altered microbiota revealed a higher percentage of secondary bile production signaling in the W–N group (Figure S5c, d).

Untargeted metabolomics was employed to investigate fecal metabolites in the W–N and N–N groups. Our analysis revealed enhanced bile secretion and primary bile acid production pathways in the W–N group, as indicated by the Kyoto Encyclopedia of Genes and Genomes (KEGG) pathway enrichment analysis (Fig. 3e). In addition, we found that DCA and taurochenodeoxycholic acid (TCDCA) were among the few metabolites with the most significant changes, and their levels were notably higher in the W–N group (Fig. 3f, g).

**Fig. 4:**
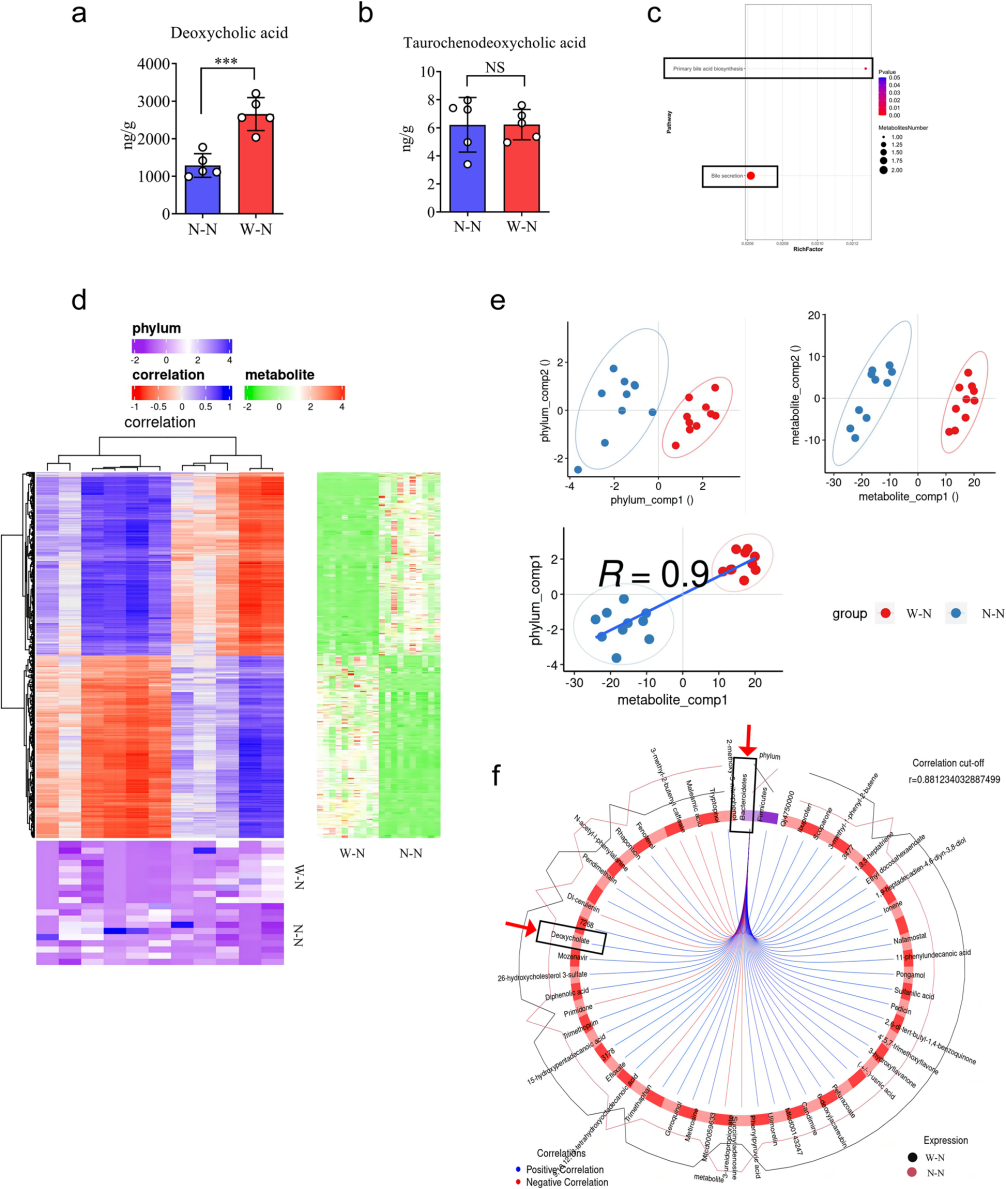
Elevated fecal DCAs are linked to an increased amount of Bacteroidetes. **a**, **b** Feces from mice in W-N and N-N groups were collected for bile acids detection through LC-MS/MS. The concentration of DCA and TCDCA in W-N and N-N groups were measured. **c** KEGG pathway enrichment analyses of the upregulated bile acids in the W-N group. The dot size represents the number of differential bile acids, and the dot color represents the corresponding *p* value. **d** (Upper left heatmap): Correlation cluster heat map of differential metabolites and microbial groups, the abscissa is microbial groups, the ordinate is differential metabolites, *P < 0.05, **P < 0.01, red means negative correlation, and blue means positive correlation. The deeper the color is, the stronger the correlation is. (Lower left heatmap): Heatmap of relative abundance of microbial groups at the phylum level. Rows represent samples, row names represent group names, and columns represent microbial groups. From purple to blue, relative abundance changes from low to high. (Upper right heatmap): Heatmap of the abundance of differential metabolites. Rows represent differential metabolites, columns represent samples, and column names represent group names. From green to red, relative abundance changes from low to high. **e** (Upper left heatmap): In the component scatter plot of the microbial group, the abscissa is the first component value, and the ordinate is the second component value; (Upper right heatmap): the component scatter diagram of differential metabolite, the abscissa is the first component value, and the ordinate is the second component value. (Lower left heatmap): Pearson correlation scatter plot of the differential metabolites and the first component of the microbiome. The vertical axis is the value of the first component of the metabolic pathway, and the horizontal axis is the value of the first component of the microbiome. A larger R indicates a higher correlation between the microbiome and the first component of the metabolic pathway. Each point in the panel represents a sample, and the colors and ellipses represent sample groups. The greater the dispersion of samples in different groups, the better the classification of that component. **f** Ring diagram of a correlation between different metabolites and microorganisms. The broken line around the ring represents the abundance of different metabolites and microbial groups in each group. The distance between the broken lines represents the difference between groups. The threshold value of the correlation coefficient is shown in the upper right corner. Only the different metabolites and microbial groups with the absolute value of correlation coefficients greater than the threshold have a line. The blue line represents positive correlations, and the red line represents negative correlations. **a**, **b** Data represent means ± SEM; NS, not significant; ***P < 0.001; by unpaired Student’s *t* test

Given the close relationship between dietary patterns, gut microbiota, and bile acid metabolism, we sought to examine if MWD could impact the bile acid metabolism of the offspring. Therefore, we utilized a high-performance liquid chromatography-tandem mass spectrometer (LC–MS/MS) to quantify the concentrations of bile acids in the feces of mice in the W–N and N–N groups, including primary BAs such as cholic acid (CA), chenodeoxycholic acid (CDCA), and secondary bile acids lithocholic acid (LCA) (Figure S6a). Our findings demonstrated that three bile acids were upregulated and three were downregulated in the W–N groups (Figure S6b, c). Furthermore, our research supported the observation that DCA was significantly more accumulated in the feces of the W–N group than in the N–N group, whereas TCDCA did not exhibit a significant change (Fig. 4a, b). KEGG pathway enrichment analyses of distinct bile acids suggested that bile secretion and primary bile acid production pathways were enhanced in the W–N group (Fig. 4c). Taken together, our results indicate that MWD alters the gut microbiota and fecal bile acids in the offspring.

### MWD-induced gut microbiota promotes elevated fecal DCA

We investigated the relationship between gut microbiota and various metabolites, as gut microbiota significantly impacts bile acid metabolism. Our findings suggested a substantial phylum-level association between the gut microbiota and fecal metabolites (Fig. 4d, e). To determine the correlation between different metabolites and microbial groups, we performed canonical correlation analysis, which employs a similarity score similar to the Pearson correlation coefficient. Our results revealed that the accumulation of fecal DCA in mice from the W–N group was positively correlated with increased *Bacteroidetes* (Fig. 4f). Additionally, the study of the gut microbiota of the W–N group at the genus levels showed an increased abundance of *Alloprevotella*, which was positively associated with increased fecal DCA levels (Figure S7a–e). Importantly, prior research has demonstrated that *Bacteroidetes* are involved in converting secondary BAs through bile salt hydrolase (BSH) activity, indicating the essential role of *Bacteroidetes* in producing DCA [32, 33].

**Fig. 5:**
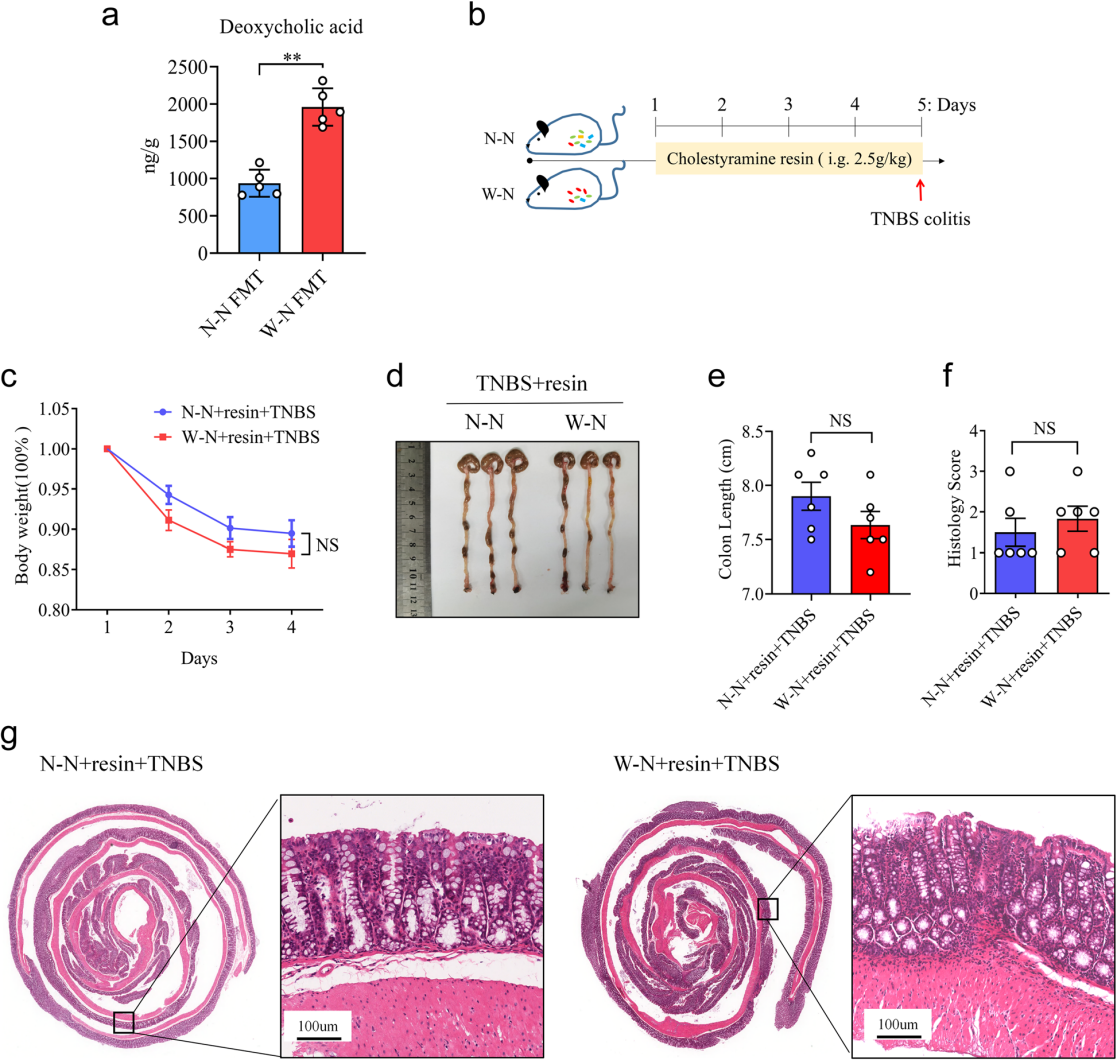
MWD promotes colitis development in their progeny by producing DCA. **a** As shown in Fig. 2a, WT mice (*n* = 5) were colonized with gut microbiota from the W-N and N-N groups. Then, the concentration of DCA in the mice’s feces was measured using an ELISA assay kit. **b**–**g** Cholestyramine resin (resin) was given to mice in the W-N and N-N groups (*n* = 6 per group) for 5 days to eliminate intestinal bile acids. Following that, mice were given TNBS to induce colitis. **c** Body weight changes in mice were evaluated daily after TNBS treatment. **d** Representative images of TNBS-treated colon in N-N+resin and W-N+resin groups. **e** The mice were sacrificed on day 4, and the colon length was recorded. **f**, **g** Histopathological analysis of colon sections. **f** Histological scores of colitis were assessed. **g** Representative images of the H&E-stained colon sections of relevant groups (scale bars 100 μm). **a**,** c**, **e**, and **f** Data represent means ± SEM; NS, not significant; **P < 0.01; by unpaired Student’s *t* test. The data shown are representative of three independent experiments

Consistently, we found that the abundance of *Bacteroidetes* was positively correlated with fecal DCA levels in both maternal and offspring mice (Figure S7f, g), indicating a potential role for MWD-derived gut microbiota in modulating DCA production. To further investigate this, antibiotics were administered to the mice in the W–N and N–N offspring groups. Notably, the synthesis of DCA was significantly reduced after *Bacteroidetes* removal, indicating that the MWD-derived microbiota is the primary driver of the elevated DCA levels (Figure S7h). Moreover, to demonstrate the impact of intestinal microbiota on DCA synthesis, wild-type FMT recipient mice were separately transplanted with microbiota from W–N and N–N mice. Importantly, mice that received W–N microbiota displayed elevated DCA levels compared to those that received N–N microbiota (Fig. 5a). These findings collectively suggest that the MWD-associated gut microbiota could increase DCA generation in offspring.

### DCA facilitates TNBS-induced colonic inflammation

To determine whether the elevated MWD-induced secondary bile acid affects the offspring’s susceptibility to colitis, mice in the W–N and N–N groups were treated with BA sequestrants, Cholestyramine resin, to remove intestinal bile acid before being challenged with TNBS (Fig. 5b). Remarkably, the deletion of BAs significantly reduced intestinal inflammation exacerbated by MWD, which failed to exacerbate clinical parameters of TNBS-induced colitis, such as body weight loss, colon shortening, and histology score (Fig. 5c–g).

**Fig. 6:**
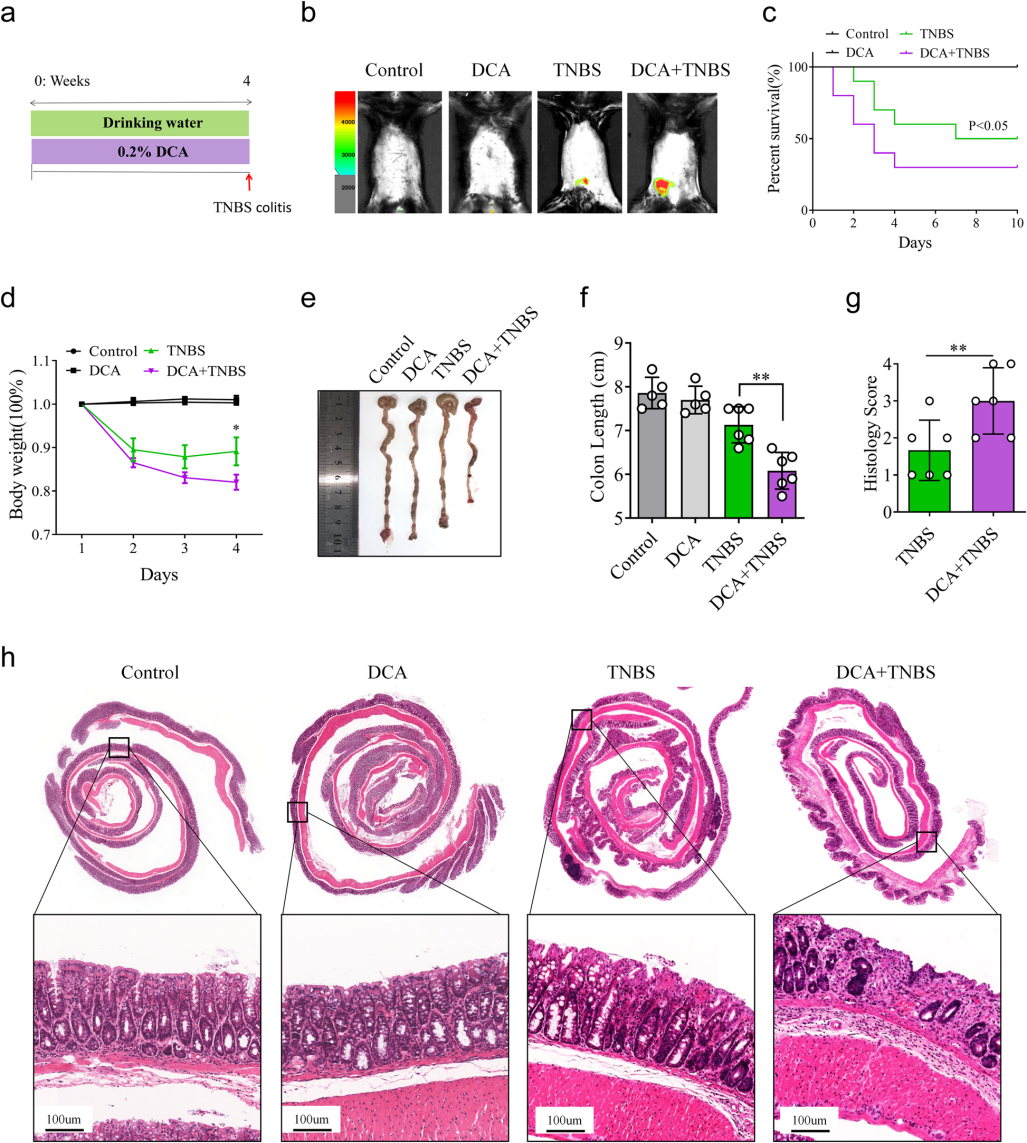
DCA facilitates TNBS-induced colonic inflammation. **a**–**h** Littermate WT mice were fed with drinking water or 0.2% DCA in drinking water for 4 weeks. Then, these mice were injected with or without TNBS via the rectal route. **b** The level of abdominal L-012 chemiluminescent signals in mice of indicated groups assessed on day 4 after TNBS treatment. **c** Survival rates of mice (*n* = 10) were monitored daily after TNBS treatment. **d** Body weight changes of mice (*n* = 5–6 per group) were monitored daily after TNBS administration. **e** Representative images of mouse colon in Control, DCA, TNBS, and TNBS+DCA groups. **f** The mice were sacrificed on day 4 after TNBS treatment, and their colon lengths were measured. **g**, **h** Colon sections were examined histologically. **g** Histology scores for colonic inflammation were measured. **h** Representative images of the H&E-stained colon sections of indicated groups (scale bars 100 μm). **c**, **d**, **f**, and **g** Data represent means ± SEM; *P < 0.05, **P < 0.01; by unpaired Student’s *t* test. The data shown are representative of three independent experiments

These findings demonstrate that MWD aggravates TNBS-induced colonic inflammation primarily by influencing bile acid metabolism and intestinal microbiota. Based on these observations, we hypothesized that DCA, the BA with the most significant variation in the W–N group, might facilitate TNBS-induced intestinal inflammation. Therefore, mice were treated with or without the secondary bile acid DCA before TNBS treatment (Fig. 6a). Notably, a single administration of DCA did not cause any epithelial damage in the colonic tissue of mice (Fig. 6b–h). However, mice treated with DCA and challenged with TNBS developed much more severe intestinal inflammation, as demonstrated by decreased survival rate, increased weight loss, and colon shortening (Fig. 6c–f). Histopathological assessments further confirmed that DCA administration aggravated TNBS-induced epithelial injury and immune cells infiltration in the colon (Fig. 6g, h). L-012, a useful compound for detecting reactive oxygen species responsible for causing inflammation, was used to determine the effects of DCA on TNBS-induced murine colitis in vivo. Our results showed that DCA administration increased the amount of abdominal L-012 chemiluminescent signals in mice treated with TNBS (Fig. 6b). Collectively, these results provide important evidence for DCA’s role in the deterioration of TNBS-induced colitis.

### DCA promotes LPS and ATP-induced pyroptosis and IL-1β secretion in macrophages

**Fig. 7:**
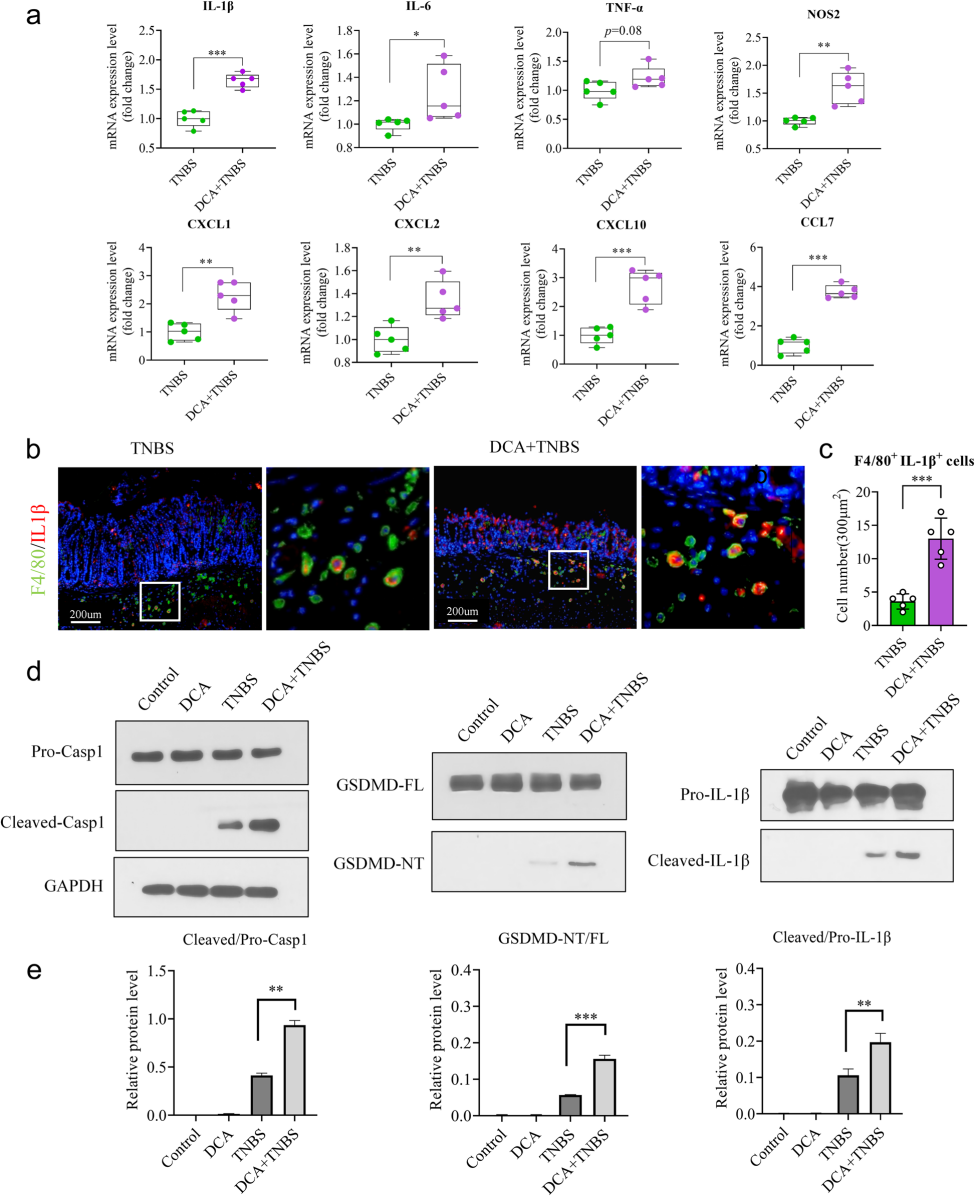
DCA intensifies TNBS-induced colitis by promoting IL-1β secretion in macrophages. **a**–**e** Littermate WT mice given or not given DCA in drinking water were treated with TNBS to induce experimental colitis, as described in Fig. 6a. On day 4, these mice were sacrificed, and their colon tissues were collected for the following analyses.** a** Relative mRNA levels of macrophages-associated cytokines (IL-1β, IL-6, TNF-α, and NOS2) and chemokines (CXCL1, CXCL2, CXCL10, and CCL7) were determined by real-time PCR and normalized to 18s. **b** Representative immunofluorescence images of F4/80 and IL-1β immunostaining in colon tissues (scale bars 200 μm). White boxes represent the magnified view.** c** The number of colonic F4/80^+^ IL-1β^+^ immune cells per 300 μm^2^. Pyroptotic cells were co-immunostained with F4/80 (green) and IL-1β (red). F4/80^+^ IL-1β^+^ cells were counted under a microscope. **d** Pro-Caspase1, Cleaved-Caspase1, GSDMD-FL, GSDMD-NT, Pro-IL-1β, Cleaved-IL-1β immunoblot images in different treatment groups. **e** Quantitative analyses of relative protein levels in different treatment groups. The density of protein bands was quantified by Alpha-view software.** a**, **c**, and **e** Data represent means ± SEM; *P < 0.05; **P < 0.01; ***P < 0.001; by unpaired Student’s *t* test. The data shown are representative of three independent experiments

Here, we measured the expression levels of pro-inflammatory cytokines and chemokines in TNBS-induced colitis. DCA administration was found to effectively increase IL-1β, IL-6, TNF-α, and NOS2 expressions in the TNBS-treated colon. Moreover, macrophage-associated chemokines, including CXCL1, CXCL2, CXCL10 and CCL7, were elevated in the colonic tissue (Fig. 7a). Hence, we hypothesized that the pro-inflammatory effect of DCA might be involved in macrophages.

Previous research has reported that DCA activates NLRP3 inflammasome and promotes IL-1β secretion in macrophages, contributing to septic shock in LPS-challenged mice [34]. GSDMD, a prominent effector typically activated by NLRP3 inflammasome, is essential for membrane pore-formation in macrophage pyroptosis, which promotes cytokine secretion and induces an immune response [35]. Thus, we proposed that DCA administration may intensify TNBS-induced colitis by regulating pro-inflammatory cytokine IL-1β secretion in macrophages.

**Fig. 8:**
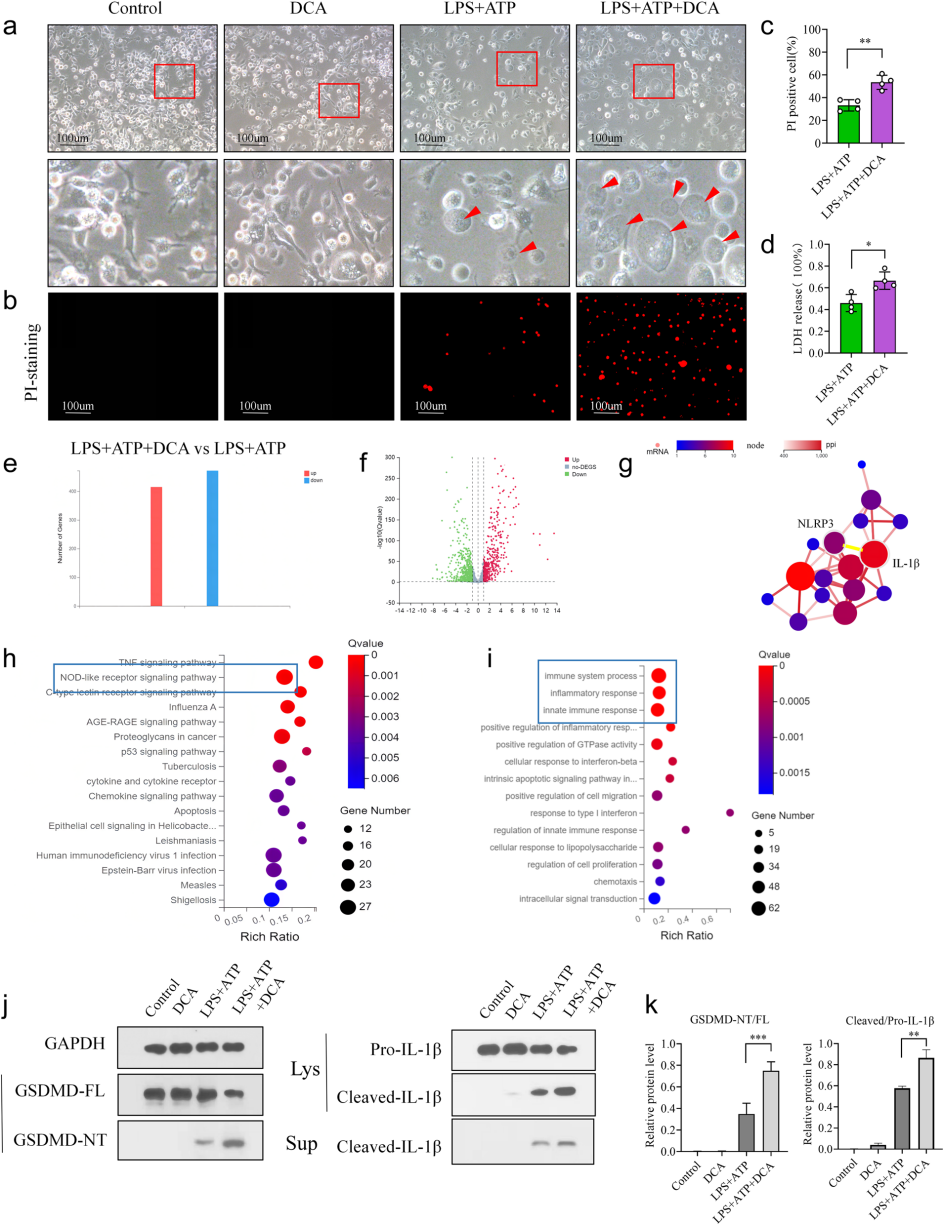
DCA promotes IL-1β secretion in LPS and ATP-stimulated macrophages. **a**–**d** Bone marrow-derived macrophages (BMDMs) isolated from mice were stimulated with LPS plus ATP or DCA alone or together. **a** Representative images of morphological changes in different treatment groups. Red boxes represent the magnified view (scale bars 100 μm). Red boxes represent the magnified view. The red arrow indicates the pyroptotic cells.** b** Representative images of PI staining in different treatment groups (scale bars 100 μm). **c** Quantitative analyses of the PI-positive cells percentage in different groups. **d** Quantitative analyses of the LDH release percentage in different groups. **e**–**i** BMDMs isolated from mice were stimulated with LPS and ATP plus DCA or not. Then, differentially expressed genes (DEGS) were analyzed by RNA sequencing.** e** The number of DEGS in the LPS and ATP plus DCA group vs. the LPS and ATP group. Red represented upregulated DEGS, and blue downregulated DEGS.** f** Scatter plot showing DEGS in the LPS and ATP plus DCA group vs. the LPS and ATP group. Genes were plotted based on their expression levels (log10 intensity). Red and green dots represented up- and downregulated genes, respectively.** g** Protein-protein interaction (PPI) analyses of the upregulated DEGs. Pyroptosis-associated genes were indicated. **h** KEGG (Kyoto Encyclopedia of Genes and Genomes) pathway enrichment analyses of the upregulated DEGs. The dot size represents the number of DEGS, and the dot color represents the corresponding *p* value.** i** GO (Gene Ontology) pathway enrichment analyses of the upregulated DEGs. The dot size represents the number of DEGS, and the dot color represents the corresponding *p* value. **j** Representative immunoblot images of GSDMD-FL, GSDMD-NT, Pro-IL-1β, and Cleaved-IL-1β in different treatment groups. **k** Relative protein levels of GSDMD-NT/FL and Cleaved-IL-1β/Pro-IL-1β in different treatment groups. The density of protein bands was quantified by Alpha-view software. **c**, **d**, and **k** Data represent means ± SEM; *P < 0.05; **P < 0.01; ***P < 0.001; by unpaired Student’s *t* test. The data shown are representative of three independent experiments

To test this hypothesis, bone marrow-derived macrophages (BMDMs) were isolated from mice and treated with DCA in vitro. We found that adding DCA induces more pyroptotic morphological changes, with membrane swelling and “bubble” formation, in LPS and ATP-treated BMDMs (Fig. 8a). Moreover, in the LPS and ATP plus DCA group, BMDMs demonstrated a higher percentage of PI-positive cells and LDH release than in the LPS and ATP group (Fig. 8b–d).

To further dissect the underlying mechanism, BMDMs isolated from mice were stimulated with LPS and ATP or with LPS and ATP plus DCA, and their mRNA expression profiles were analyzed by RNA sequencing. We observed that a large number of genes, including pyroptosis-associated genes such as Jun, Plcb3, IL-1β, NLRP3, Rela, and Mefv, were upregulated in the LPS and ATP plus DCA group compared to the LPS and ATP group (Fig. 8e, f). KEGG and GO (Gene Ontology) pathway enrichment analysis showed that these upregulated genes were significantly enriched in regulating the NOD-like receptor signaling pathway and inflammatory response signaling pathway, respectively (Fig. 8h, i). Moreover, protein–protein interaction network analysis identified NOD-like receptor thermal protein domain-associated protein 3 (NLRP3) as an upstream signaling effector of IL-1β (Fig. 8g). Furthermore, DCA addition effectively led to the increased protein expression levels of GSDMD-N-terminal (GSDMD-NT) and cleaved IL-1β in LPS and ATP-treated BMDMs, but not in untreated BMDMs (Fig. 8j, k). Overall, these results suggested that DCA could directly promote GSDMD-mediated pyroptosis and IL-1β secretion in macrophages.

### DCA exacerbates colitis by triggering GSDMD-dependent pyroptosis and IL-1β secretion

Pyroptosis plays a crucial role in driving the inflammatory response, but it remains unclear whether GSDMD-mediated pyroptosis contributes to the pathogenesis of Crohn’s disease (CD). To investigate this, we evaluated the expression of F4/80, IL-1β, and GSDMD-NT in colonic tissues obtained from CD patients. Our findings demonstrate a significant increase in F4/80-positive cells in the colon of active CD patients compared to those in remission and healthy controls. Notably, we observed co-localization of GSDMD-NT and IL-1β in F4/80-positive cells, indicating that GSDMD-mediated pyroptosis and IL-1β secretion in macrophages may be involved in CD disease activity (Figure S8a, b).

**Fig. 9:**
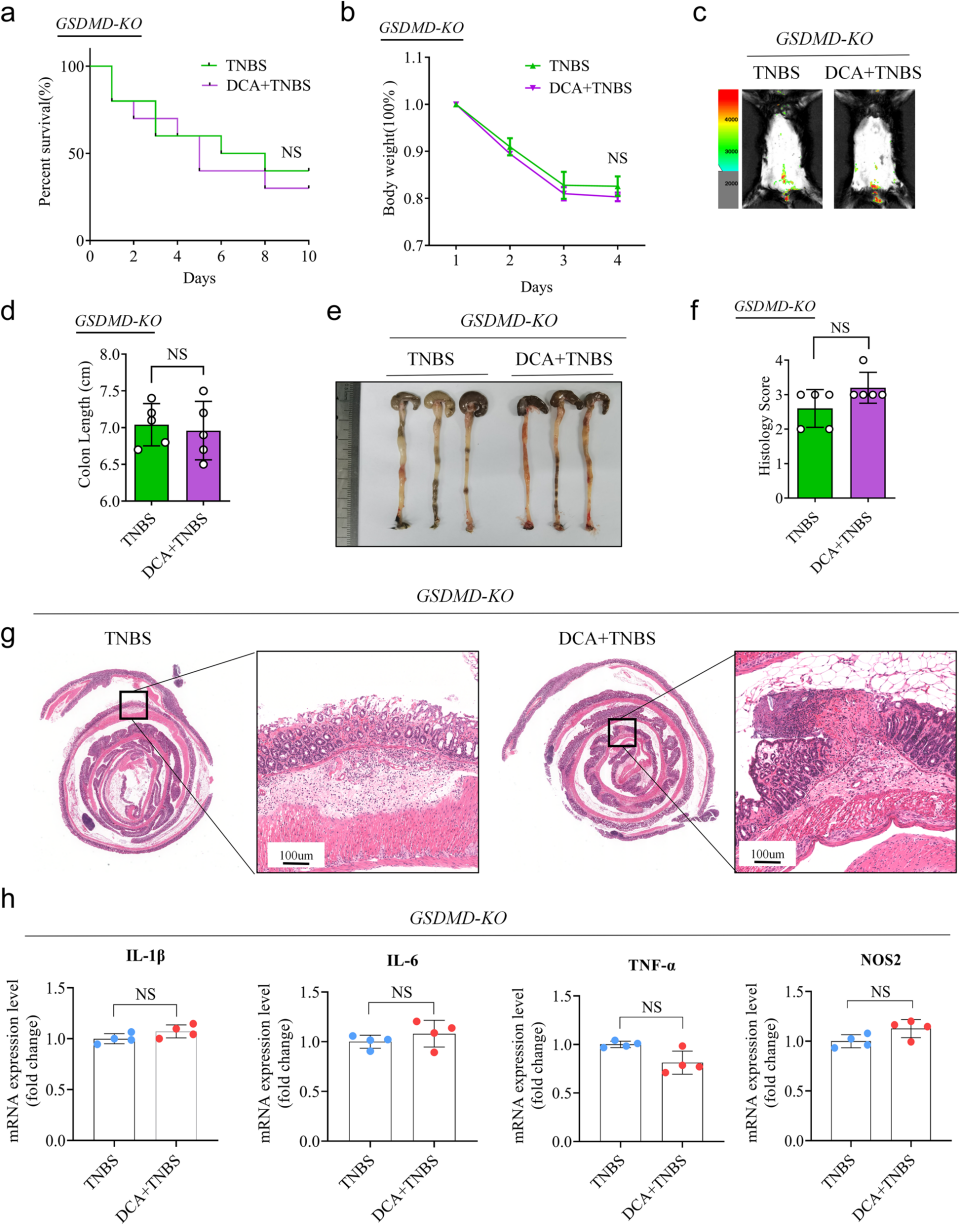
DCA medicated GSDMD dependent pro-inflammatory effect on TNBS-induced colitis. **a**–**h** Littermate *GSDMD-KO* mice given or not given DCA in drinking water were treated with TNBS to induce experimental colitis, as described in Fig. 6a. On day 4, these mice were sacrificed, and their colon tissues were collected for the following analyses. **a** Survival rates of mice (*n* = 10) were monitored daily after being administrated with TNBS solution. **b** Body weight changes of mice (*n* = 5) were monitored daily after TNBS administration. **c** Level of abdominal L-012 chemiluminescent signals in mice treated with TNBS were assessed on day 4 after TNBS treatment. **d** Mice were sacrificed on day 4 after TNBS treatment, and their colon lengths were measured. **e** Representative images of mouse colon in TNBS, TNBS+DCA groups. **f**, **g** Colon sections were histologically assessed.** f** Histology scores for colonic inflammation were measured. **g** Representative images of the H&E-stained colon sections of indicated groups (scale bars 100 μm). **h** Relative mRNA levels of pro-inflammatory cytokines IL-1β, IL-6, TNF-α, and NOS2 were determined by real-time PCR as normalized to 18s. **a**, **b**, **d**, **f**, and **h** Data represent means ± SEM; NS, not significant; by unpaired Student’s *t* test. The data shown are representative of three independent experiments

As expected, the administration of DCA resulted in a significant increase in the expression of Cleaved-Caspase1, Cleaved-IL-1β and GSDMD-NT in TNBS-treated colon tissues (Fig. 7d, e). Furthermore, DCA administration significantly increased the number of F4/80^+^ IL-1β^+^ macrophages (Fig. 7b, c). Since GSDMD cleavage is crucial for inducing pyroptosis and IL-1β secretion in macrophages, we hypothesized that it also contributes to promoting intestinal inflammation by DCA. We tested this hypothesis by administering DCA to *GSDMD-KO* mice and induced colitis using TNBS. The results showed that deletion of GSDMD significantly mitigated the effects of DCA on TNBS-induced colitis, as reflected by no significant differences in survival rate, weight loss, colonic length shortening and histology scores between the TNBS plus DCA and TNBS groups (Fig. 9a-g). Moreover, the deletion of GSDMD largely diminished the differential expression levels of inflammatory markers in mice (Fig. 9h). Collectively, these results suggest that DCA exacerbates TNBS-induced colitis by activating GSDMD-dependent pyroptosis and IL-1β secretion.

## Discussion

Epidemiological studies have shown a link between WD patterns and an increased likelihood of developing CD [36]. Long-term ingestion of WD can negatively affect gut homeostasis by disturbing intestinal microbiota, causing Paneth cell dysfunction and mucus barrier disruption, all of which can lead to increased susceptibility to infection and IBD [6, 26, 37, 38]. Mice fed a high-fat and high-sugar western diet had higher DCA levels, which led to the malfunctioning of intestinal Paneth cells, a key role in the pathogenesis of CD [26, 39]. However, despite the association between WD consumption and a higher risk of CD, clinical patients with CD are rarely overweight or obese [40]. From another perspective, maternal factors such as dietary intake or obesity, breastfeeding, gut microbiota, and environmental exposures can potentially alter the offspring’s health during pregnancy and long after delivery [31, 41, 42]. Thus, we hypothesized that maternal factors might contribute to the incidence of CD influenced by WD consumption.

Early colonization of the infant’s gut with microbes plays a critical role in the development of immunity and metabolic function. A recent study showed that dysbiotic microbiota in 2-week-old infants born to obese mothers with a pre-pregnancy BMI over 30 induced metabolic and inflammatory changes in the liver and bone marrow cells of germ-free mice colonized with this dysbiotic microbiota [18]. Additionally, mice colonized with fecal samples from 2-week-old newborns born to obese mothers had a lower *Bacteroidetes*/*Firmicutes* ratio, consistent with an obese animal model. In our study, offspring born to WD-fed mothers (the W–N group) had a higher abundance of *Bacteroidetes* and a lower level of *Firmicutes*, similar to the altered gut microbiota observed in CD patients [43].

Our findings reveal that maternal consumption of a WD exacerbates TNBS-induced colitis in offspring, likely due to altered intestinal microbiota and related metabolites. However, the mechanisms via which the maternal effects influence the gut microbiota of offspring remain unclear. Recent research has shown that mouse offspring derived from oocytes of maternal hyperglycemia display glucose intolerance, highlighting the potential impact of maternal oocyte development on the prognosis of diseases in the next generation [44]. Furthermore, it is noteworthy that epigenetic alterations in offspring may also affect the gut microbiota [45–47]. In this study, we evaluated the mucus layer, intestinal barrier integrity and colonic histology, all closely related to gut homeostasis and microbiota but were not found to have significant effects of maternal influence on these factors in offspring. However, further genomic studies focusing on histone alterations and DNA methylation are needed. Additionally, the offspring mice were nurtured by their mothers until weaning, suggesting that altered gut microbiota could be due to breastfeeding factors [48, 49]. However, a cross-feeding experiment may be required to prove this suspicion.

BA metabolism is strongly correlated with gut microbiota and dietary patterns [50]. In this study, we initially identified an increased fecal production of DCA in the W–N group of mice compared to the N–N group, using untargeted metabolomics analysis. Based on previous studies, the conversion of bile acids by specific intestinal bacteria is directly linked to serum and fecal bile acid composition [50]. Notably, correlation analysis of microbiota and metabolites showed that increased DCA levels were strongly related to the abundance of *Bacteroidetes* phylum, which significantly accumulated in the W–N group. *Bacteroidetes,* a group of gut commensal microbiota with specific bile salt hydrolase (BSH) activity, have been shown to participate in the generation of DCA [51, 52]. Therefore, increased *Bacteroidetes* proportions have been correlated with increased BSH activity, which successfully deconjugates BAs attached to taurine or glycine by host liver enzymes, revealing unconjugated bile acids. This deconjugation occurs before bacteria convert primary bile acids to secondary bile acids [24]. In the present study, we also observed a significantly decreased level of glycocholic acid (GCA) but no significant changes in taurocholic acid (TCA) and CA (Figure S6a–c). These results suggest that BSH-expressing *Bacteroidetes* likely deconjugated GCA to generate unconjugated CA, which was then converted to DCA in subsequent bacterial conversion processes. This partly explains the elevated DCA levels observed in the W–N group mice. Importantly, fecal transplantation experiments in this study supported the elevated DCA in relevance to altered intestinal microbiota in the W–N group. Eliminating the microbiota effectively reduced the increased DCA production in the gut. These findings provide crucial support for the notion that the microbiota plays an indispensable role in DCA generation and the progression of colitis. However, although both pre- and post-weaning exposure to WD increased offspring susceptibility to colitis, we did not further compare the microbiota’s contribution to this phenotype using fecal microbiota transplantation (FMT). Further research is needed to distinguish the role of maternal and offspring diet in influencing the microbiota.

The etiology of numerous autoimmune illnesses, including IBD, has been linked to interleukin-1β (IL-1β) released by macrophages in response to lipopolysaccharide (LPS) [53]. Colonic mucosa samples from IBD patients have been shown to contain more IL-1β^+^ macrophages and monocytes than healthy controls [54]. Increased IL-1β production results in inflammatory cell activation and chemotaxis, which contribute to the development of intestinal inflammation [55]. Additionally, GSDMD has pyroptotic activity due to the pore-forming activity of its N-terminal domain, which is cleaved by activated inflammatory caspases associated with IL-1β release [56]. Therefore, understanding how IL-1β is triggered and secreted by macrophages is crucial for treating chronic immunological problems in IBD. In this study, we discovered that DCA administration exacerbates TNBS-induced colitis. Moreover, in TNBS-exposed colons, DCA administration resulted in significantly higher expression of GSDMD-NT and cleaved IL-1β. In vitro experiments demonstrated that DCA could directly accelerate LPS and ATP-induced pyroptosis in bone marrow-derived macrophages (BMDMs), as DCA dramatically boosted GSDMD cleavage and IL-1β release, although DCA administration alone had negligible effect. Furthermore, knocking down GSDMD in mice considerably eliminated DCA’s pro-inflammatory action in experimental colitis. These findings suggest that DCA aggravates TNBS-induced colitis in macrophages by regulating GSDMD-dependent pyroptosis and IL-1β production.

The major effector, GSDMD, must be fully cleaved by classical inflammasome signaling or non-canonical activation of caspase11 to induce pyroptotic cell death [57, 58]. In macrophage responses to cytoplasmic lipopolysaccharide or Gram-negative bacteria, non-canonical activation of caspase11 is commonly reported [59, 60]. The canonical activation of inflammatory caspase1 generally requires the formation of the NLRP3 inflammasome, which is crucial for the maturation of interleukin-1β/IL-18 and is triggered by various microbial pathogens and small molecular metabolites [61, 62]. Intriguingly, a recent study found that elevated levels of DCA activated the NLRP3 inflammasome in macrophages [63]. The underlying mechanism of DCA triggering the NLRP3 inflammasome is thought to be the activation of the S1PR2-cathepsin B pathway. These findings could explain the increased GSDMD cleavage and IL-1β release in our model. However, further research is needed to determine whether the NLRP3 inflammasome plays a role in this process. It is also unclear whether DCA induces pyroptotic cell death in macrophages via the farnesoid X receptor, G-protein coupled receptor, or other host nuclear receptors.

Paneth cell dysfunction, a key factor in IBD gut inflammation, was previously observed in the small intestine of mice fed a high-fat, high-sugar Western diet [26]. Moreover, WD ingestion increased *Clostridium*, a bacterium that mediates the conversion of the secondary bile acid DCA. Further research has shown that DCA promotes Paneth cell dysfunction by activating the farnesoid X receptor (FXR) and type I interferon (IFN) signaling pathways. These findings suggest that the increased activation of FXR and type I IFN caused by DCA is closely related to the WD-associated Paneth cell dysfunction [26]. This raises the possibility that MWD-associated DCA might also impair Paneth cells, contributing to the development of colitis. Although mounting evidence supports the correlation between IBD onset and WD intake, in this study, we only used a single model to explore maternal influences. Other models, including DSS or chronic colitis, require further investigation into this phenotype and its underlying mechanisms in the future.

## Conclusion

Overall, our study revealed that maternal WD could alter the intestinal microbiota of offspring, which in turn affects intestinal bile acid metabolism. Based on our findings, we propose a hypothesis for the effect of maternal Western diet exposure on offspring’s susceptibility to CD-like colitis (Figure S9). Maternal consumption of a Western-style high-fat, high-sugar diet leads to increased levels of *Bacteroidetes*, which express selective bile salt hydrolase (BSH) activity and contribute to the generation of unconjugated bile acid and secondary bile acid DCA in offspring. MWD-associated DCA promotes GSDMD-mediated pyroptotic cell death and IL-1β release in macrophages, resulting in increased intestinal inflammation generated by TNBS treatment. Therefore, our study reveals a possible link between maternal WD intake and the risk of offspring developing CD.

## Supplementary Information

**Additional file 1:** **Table S1.** Dietary compositions and caloric contents of high fat purified rodent diet. **Table S2.** Dietary compositions and caloric contents of control diet. **Table S3.** Demographic characteristics of the study population. **Table S4.** Primers for RT-PCR.

**Additional file 2:** **Figure S1.** Female mice exposed to WD develop a metabolic abnormality. **Figure S2.** Dietary intervention does not affect the tight junctions of the colon. **Figure S3.** Post-weaning WD consumption disrupts the mucus barrier in offspring. **Figure S4.** MWD facilitates offspring susceptibility to TNBS-induced colitis in a gut microbiota-dependent manner. **Figure S5.** Composition and function prediction of microbiota affected by MWD. **Figure S6.** MWD affects bile acid metabolism in offspring. **Figure S7.** Altered gut microbiota is linked to elevated DCA levels. **Figure S8.** GSDMD-medicated pyroptosis and IL-1β secretion are involved with CD development. **Figure S9.** Model showing how maternal Western diet influences offspring’s vulnerability to TNBS-induced CD-like colitis.

## Data Availability

The data sets supporting the conclusions of this article are listed as follows: http://bio-annotation.cn/gutmgene/search.dhtml. The raw metagenome sequencing data of 16S rDNA reported in this paper have been deposited in the Genome Sequence Archive database (accession no. CRA010083). The raw metagenome sequencing data of RNA-seq reported in this paper have been deposited in the Genome Sequence Archive database (accession no. CRA010081). The raw data of Non-Targeted Metabolomics reported in this paper have been deposited in the OMIX database (accession no. OMIX003250).

## Abbreviations

IBD: Inflammatory Bowel Disease
CD: Crohn's disease
UC: Ulcerative Colitis
TNBS: Trinitrobenzenesulfonic acid solution
WD: Western-style diet
MWD: Maternal Western diet
ND: Normal control diet
IOD: Integrated optical density
NLRP3: NOD-like Receptor Protein 3
ZO: Zonula Occludens
OCLN: Occludin
BMDMs: Bone Marrow-derived Macrophages
LPS: Lipopolysaccharide
FMT: Fecal microbiota transplantation
H&E: Hematoxylin-eosin
MUC2: Mucin2
PAS: Periodic Acid-Schiff
WAT: White adipose tissue
ATP: Adenosine Triphosphate
GSDMD: Gasdermin D
GSDMD-NT: GSDMD-N-terminal
GSDMD-FL: GSDMD-full-length
DCA: Deoxycholic Acid
CA: Cholic Acid
TCDCA: Taurochenodeoxycholic acid
LCA: Lithocholic acid
FXR: Farnesol X receptors
TGR5: G protein-coupled bile acid receptor
DSS: Dextran sodium sulfate
