# Supplementary material for: Maternal Western diet mediates susceptibility of offspring to Crohn’s-like colitis by deoxycholate generation
Source: Microbiome. 2023 May 2;11:96. doi: 10.1186/s40168-023-01546-6 (PMC10155335; doi:10.1186/s40168-023-01546-6)
Supplement: Supplementary file 2 — Additional file 1: Table S1. Dietary compositions and caloric contents of high fat purified rodent diet. Table S2. Dietary compositions and caloric contents of control diet. Table S3. Demographic characteristics of the study population. Table S4. Primers for RT-PCR. [file 40168_2023_1546_MOESM1_ESM.docx]

**Maternal Western diet mediates susceptibility of offspring to Crohn’s-Like colitis by deoxycholate generation**

**Chongyang Huang**^1, 2, †^, **Huishi Tan**^3, †^, **Mengyao Song**^1, †^, **Ke Liu**^1, †^, **Hongbin Liu**^1^, **Jun Wang**^2^, **Yanqiang Shi**^4^, **Fengyi Hou**^1^, **Qian Zhou**^1^, **Ruo Huang**^1^, **Binghai Shen**^1^, **Xinlong Lin**^1^, **Xiaoming Qin**^1^, **Fachao Zhi**^1, *^

^1^Guangdong Provincial Key Laboratory of Gastroenterology, Institute of Gastroenterology of Guangdong Province, Department of Gastroenterology, Nanfang Hospital, Southern Medical University, Guangzhou, China

^2^Department of Gastroenterology, The Second Affiliated Hospital of Guangzhou University of Chinese Medicine, Guangzhou, China

^3^State Key Laboratory of Organ Failure Research, National Clinical Research Center of Kidney Disease, Division of Nephrology, Nanfang Hospital, Southern Medical University, Guangzhou, China

^4^Institute of Dermatology and Venereology, Dermatology Hospital, Southern Medical University, Guangzhou, China; Department of Science & Education, Dermatology Hospital, Southern Medical University, Guangzhou, China.

^†^These authors contributed equally to this study

^*^Correspondence and requests for materials should be addressed to F.Z. (zhifc41532@163.com)


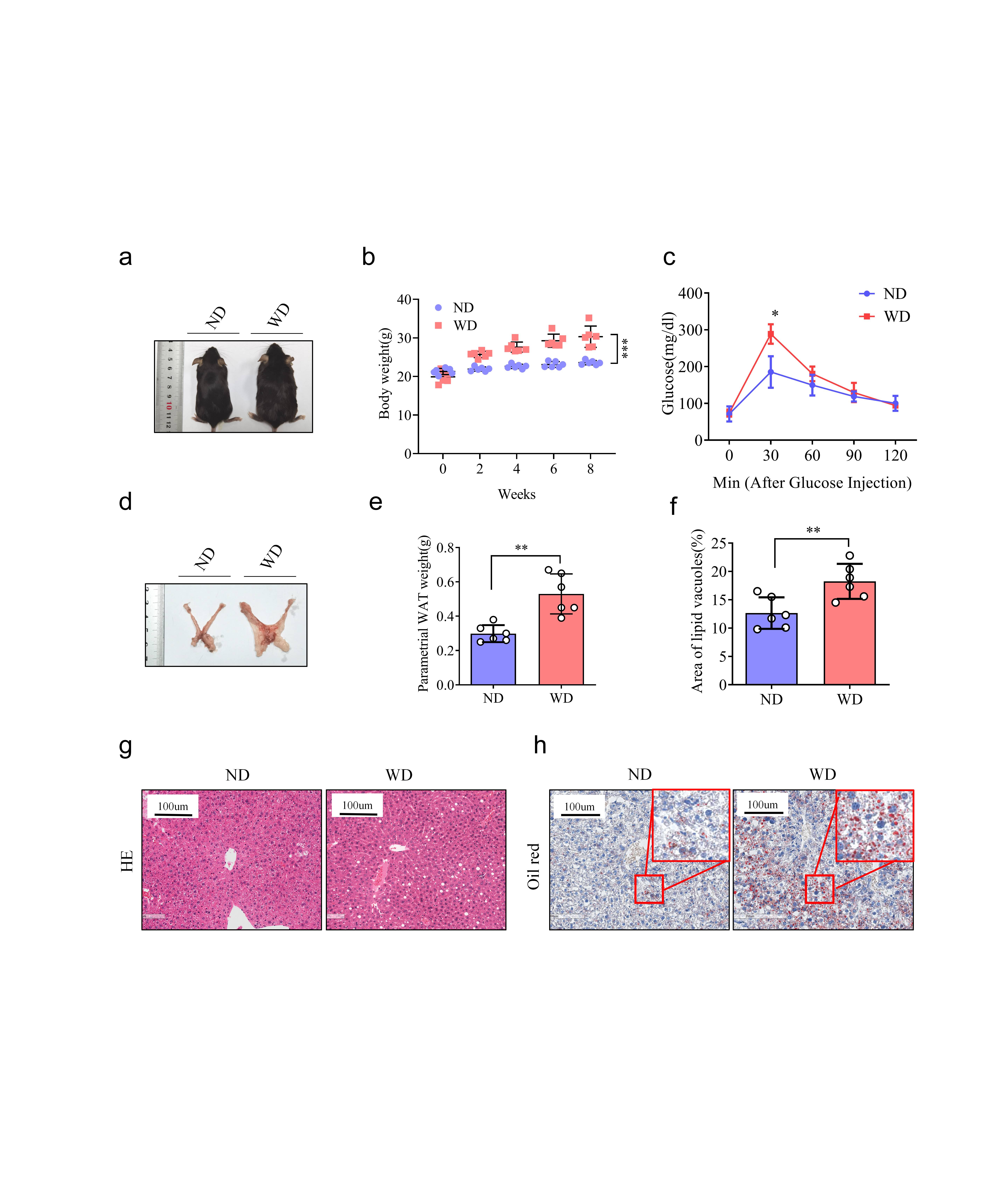


**Figure S1 Female mice exposed to WD develop a metabolic abnormality**

(a to h) Female WT mice were fed a Western diet (WD) or normal diet (ND) for 8 weeks and assessed for metabolic status, including weight gain, glucose tolerance, white adipose weight, and liver lipid accumulation.

1. The appearance of mice fed on ND and WD.
2. Body weight changes of mice fed on ND and WD.
3. Glucose tolerance in mice fed on ND and WD.
4. Representative images of parametrial white adipose tissue(WAT) in mice.
5. Quantitative analyses of parametrial WAT weight in mice on ND and WD.

(f to h) Liver tissues were examined by H&E and Oil red staining.

1. Quantitative analyses of percentage area with lipid vacuoles in the liver.
2. Representative images of the H&E-stained liver tissue of indicated groups.
3. Representative images of the Oil red-stained liver tissue of indicated groups.

(b, c, e and f) Data represent means ± SEM (n = 5 per group); **P* < 0.05; ***P* < 0.01; ****P* < 0.001; by unpaired Student’s *t*-test. The data shown are representative of three independent experiments.


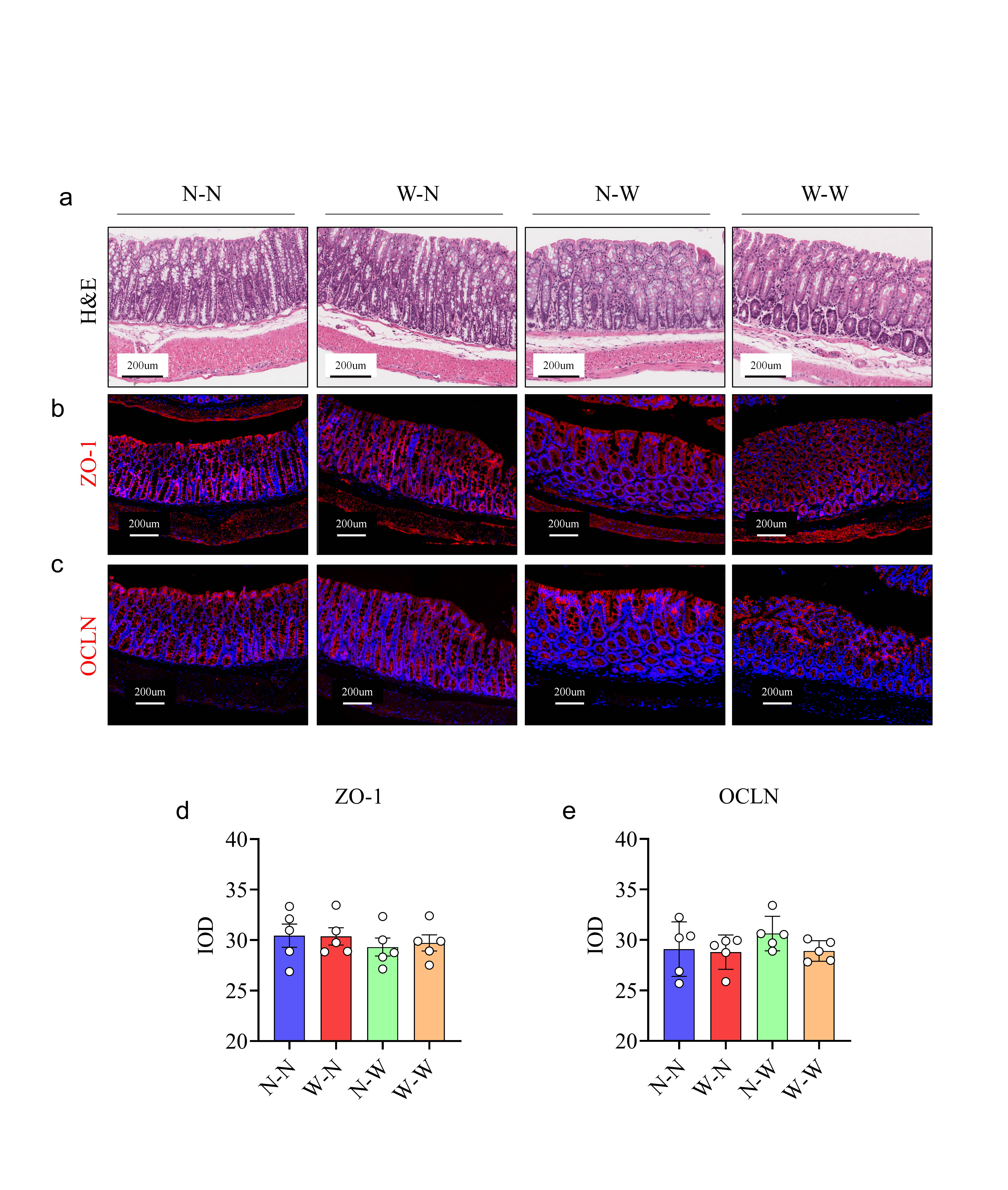


**Figure S2 Dietary intervention does not affect the tight junctions of the colon**

(a to e) Mice from indicated groups were sacrificed at the age of 8 weeks and their colons were collected for H&E staining and immunostaining analyses.

1. Representative images of the H&E-stained colon sections of different treatment groups (scale bars: 200 μm).

(b and c) Representative immunofluorescence images of ZO-1 and Occludin (OCLN) immunostaining in colon tissues of different treatment groups (scale bars: 200 μm).

(d and e) Quantitative analyses of ZO-1 and OCLN staining by Image-Pro Plus software. IOD: integrated optical density.

Data represent means ± SEM (n = 5 per group); NS, not significant; Unpaired Student’s *t*-test was performed for statistical analysis. The data shown are representative of two independent experiments.


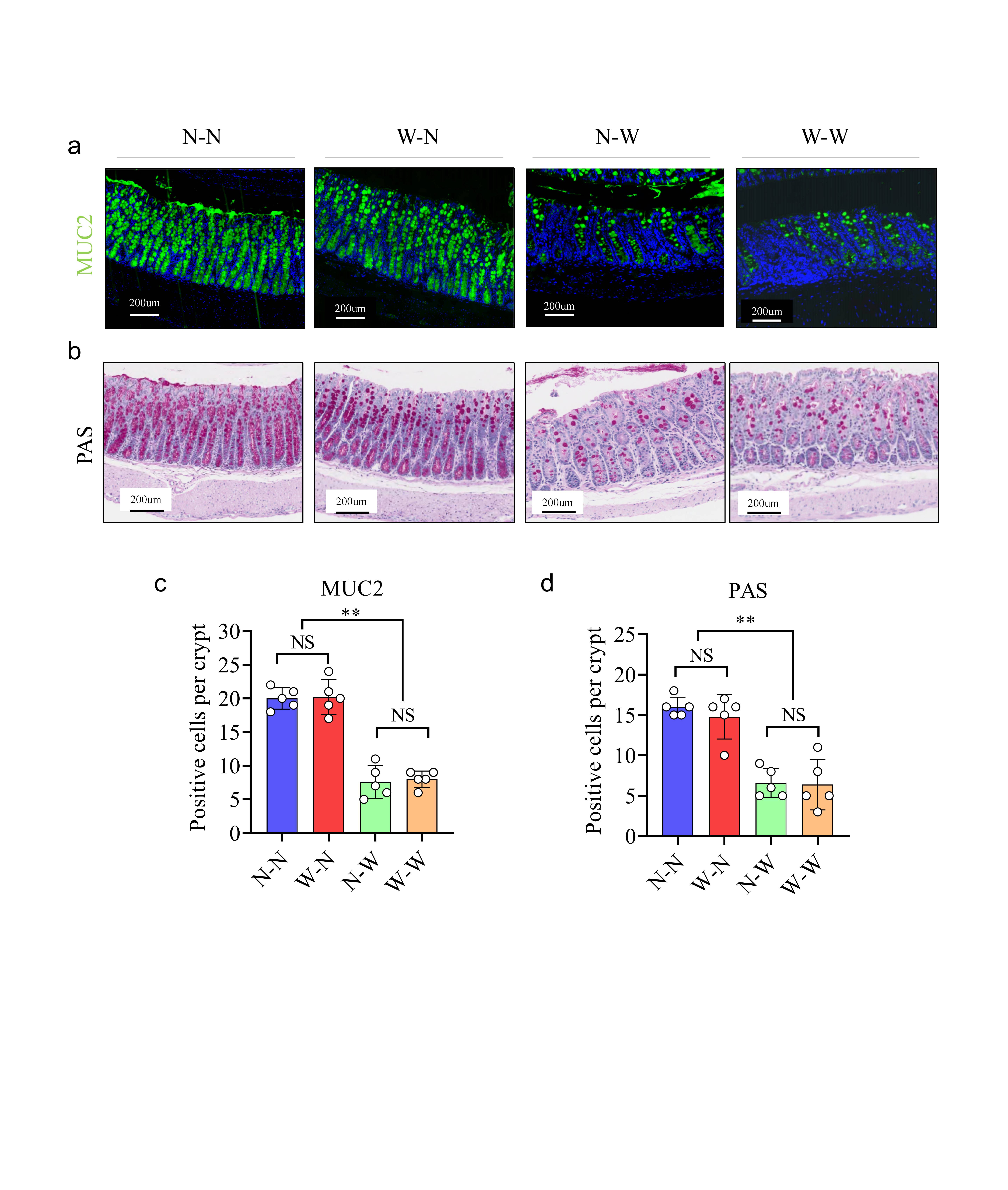


**Figure S3 Post-weaning WD consumption disrupts the mucus barrier in offspring**

(a to d) Mice from indicated groups were sacrificed at the age of 8 weeks and their colons were collected for PAS staining and immunostaining analyses.

(a and b) Representative immunofluorescence images of mucin 2 (MUC2) and PAS immunostaining in colon tissues (scale bars: 200 μm).

(c and d) Quantitative analyses of MUC2 and PAS staining by Image-Pro Plus software. IOD: integrated optical density.

Data represent means ± SEM (n = 5 per group); ***P* < 0.01; NS, not significant; Unpaired Student’s *t*-test was performed for statistical analysis. The data shown are representative of two independent experiments.


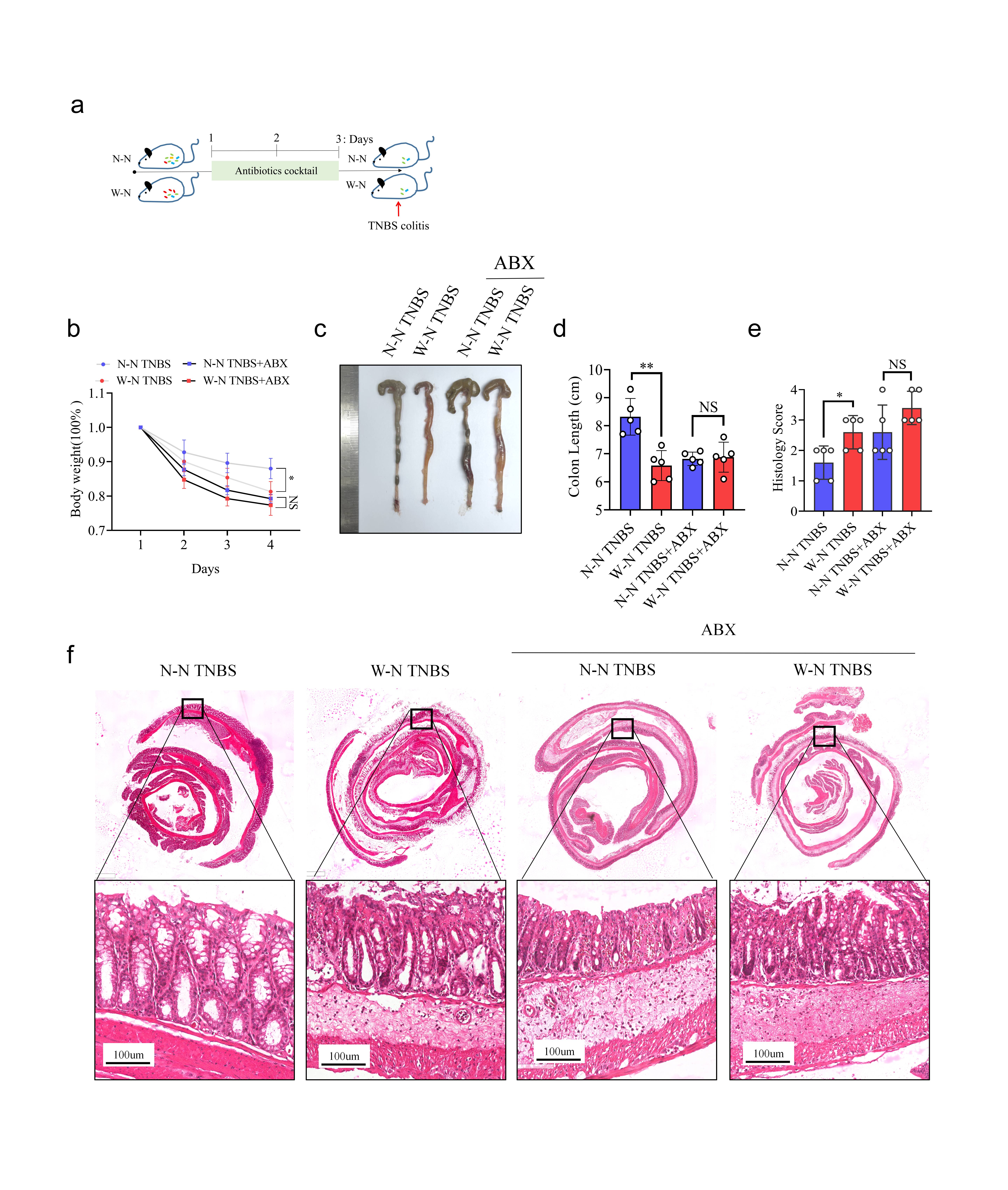


**Figure S4 MWD facilitates offspring susceptibility to TNBS-induced colitis in a gut microbiota-dependent manner**

(a to f) Mice in W-N and N-N groups were administered antibiotics for 3 days to remove gut microbiota, then injected with TNBS to induce colitis via the rectum

(b) Body weight changes of mice were monitored daily after TNBS administration.

(c) Representative images of the colon of mice treated with TNBS in the N-N, W-N, N-N+ABX and W-N+ABX groups.

(d) The mice were sacrificed on day 4 after TNBS treatment, and their colon lengths were measured.

(e and f) Colon sections were examined histologically.

(e) Histology scores for colonic inflammation were measured.

(f) Representative images of the H&E-stained colon sections of relevant groups (scale bars: 100 μm).

(b, d, and e) Data represent means ± SEM (n = 5 per group); NS, not significant; **P*< 0.05; ***P*< 0.01; by unpaired Student’s *t*-test.


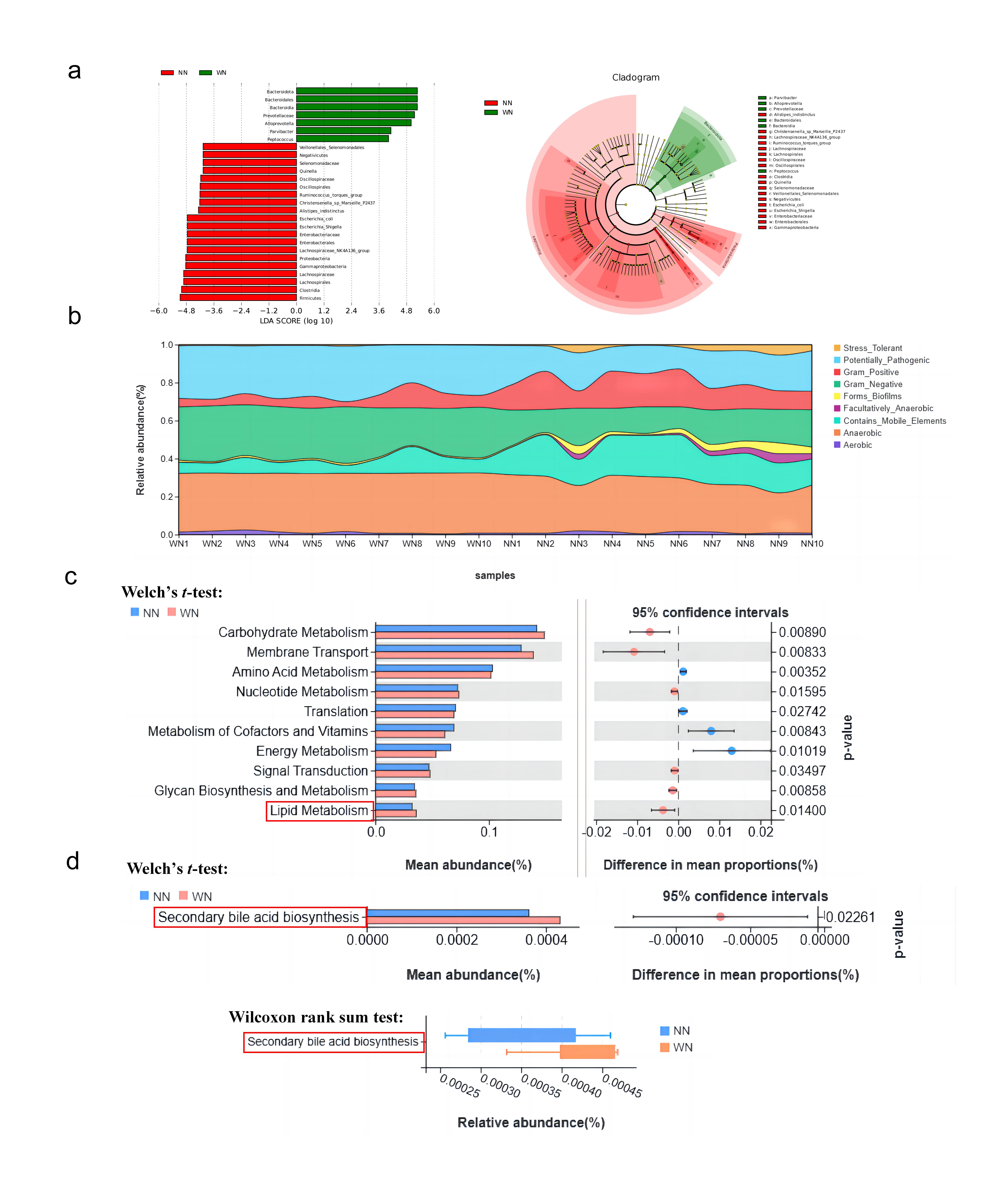


**Figure S5 Composition and function prediction of microbiota affected by MWD**

(a to d) The intestinal microbiota of mice in the W-N and N-N groups were analyzed for differential composition and potential function.

1. Column diagram and Cladogram show differential microbiota according to LEfse analyses between the W-N and N-N groups. The x-axis shows Linear Discriminant Analysis (LDA) scores. Microbiota with significant variation is colored according to the group, with red nodes indicating microbial taxa that play a key role in the N-N group, and green nodes indicating microbial taxa that play an important role in the W-N group.
2. BugBase predicts the microbiota’s functional phenotype, including Stress tolerance, Biofilm formation, Pathogenicity, Gram-positive, Gram-negative, Mobile elements, Oxygen demand, Anaerobic, Aerobic, and Facultatively anaerobic bacteria.
3. Tax4Fun function prediction with differential microbiota, showing a relative functional abundance in KEGG signaling (Level 2).
4. Tax4Fun function prediction with differential microbiota, showing a relative functional abundance in Secondary bile acid synthesis signaling (Level 3).

Data represent means ± SEM (n = 10 per group); Welch’s *t*-test and Wilcoxon rank sum test were performed for statistical analysis. The data shown are representative of two independent experiments.


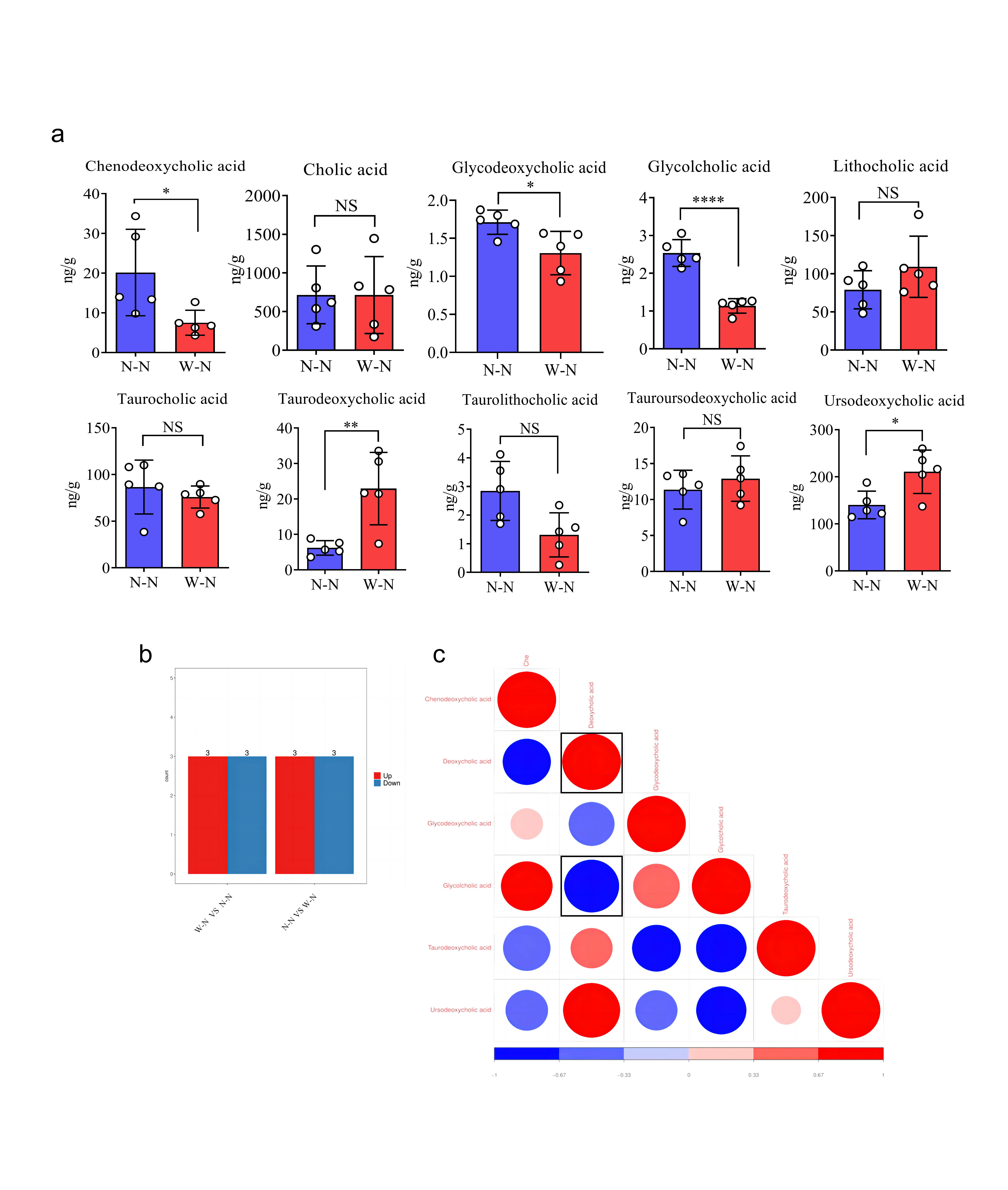


**Figure S6 MWD affects bile acid metabolism in offspring**

(a to c) Feces were collected from mice in W-N and N-N groups and detected for bile acid concentration through LC-MS/MS.

1. Quantitative fecal bile acid concentration analyses in mice from W-N and N-N groups.
2. Statistical bar plot of differential metabolites. The abscissa represents the comparison group, and the ordinate represents the number of differential metabolites. Red represents the number of up-regulated metabolites, and blue represents the number of down-regulated metabolites.
3. Correlation heat map of differential metabolites. The abscissa represents the metabolite name, and the ordinate represents the corresponding metabolite name. Red indicates a positive correlation, blue indicates a negative correlation, and the deeper the color is, the stronger the correlation is.

Data represent means ± SEM (n = 5 per group); NS, not significant; **P* < 0.05; ***P* < 0.01; *****P* < 0.0001; Unpaired Student’s *t*-test was performed for statistical analysis. The data shown are representative of two independent experiments.


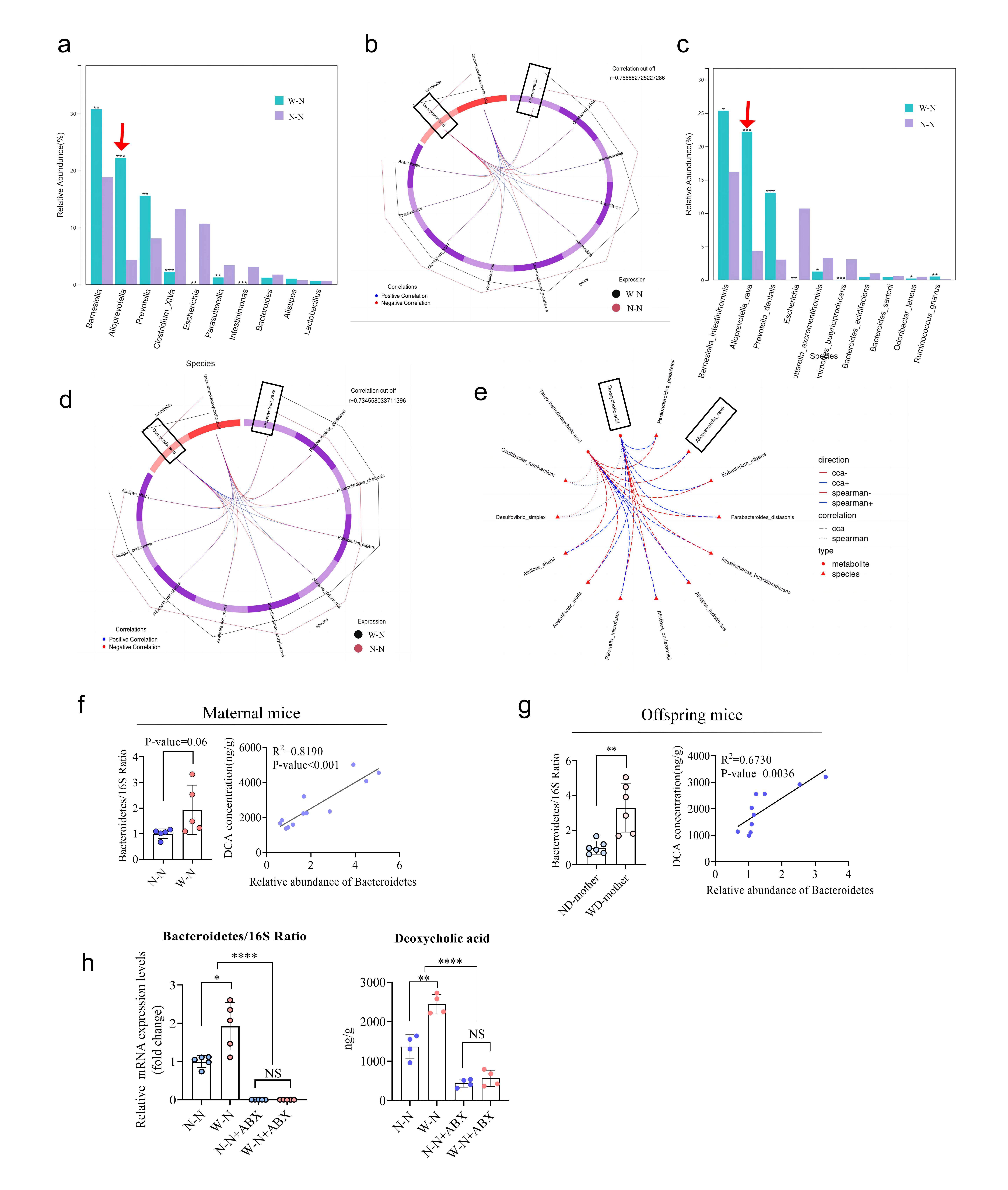


**Figure S7 Altered gut microbiota is linked to elevated DCA levels**

(a) Comparative strain analysis at the genus level.

(b) At the genus level, correlation analysis of DCA and differential strains.

(c) Comparative strain analysis at the species level.

(d) At the species level, correlation study of DCA and differential strains.

(e) Network diagram depicting the relationship between various metabolites and microbial groupings.

(f) Relative abundance of *Bacteroidetes* in maternal mice of ND-mother and WD-mother groups were determined by real-time PCR, and further correlated with fecal DCA levels.

(g) Relative abundance of *Bacteroidetes* in offspring mice of W-N and N-N groups were determined by real-time PCR, and further correlated with fecal DCA levels.

(h) Mice (n = 4) in W-N and N-N groups were administered antibiotics for 3 days to remove gut microbiota. The relative abundance of *Bacteroidetes* in mice were determined by real-time PCR. Then, using an ELISA assay kit, the concentration of DCA in the mice's feces was determined.

Data represent means ± SEM; NS, not significant; *P < 0.05; **P < 0.01; ***P < 0.001; ****P < 0.0001; Unpaired Student’s *t*-test was performed for statistical analysis.


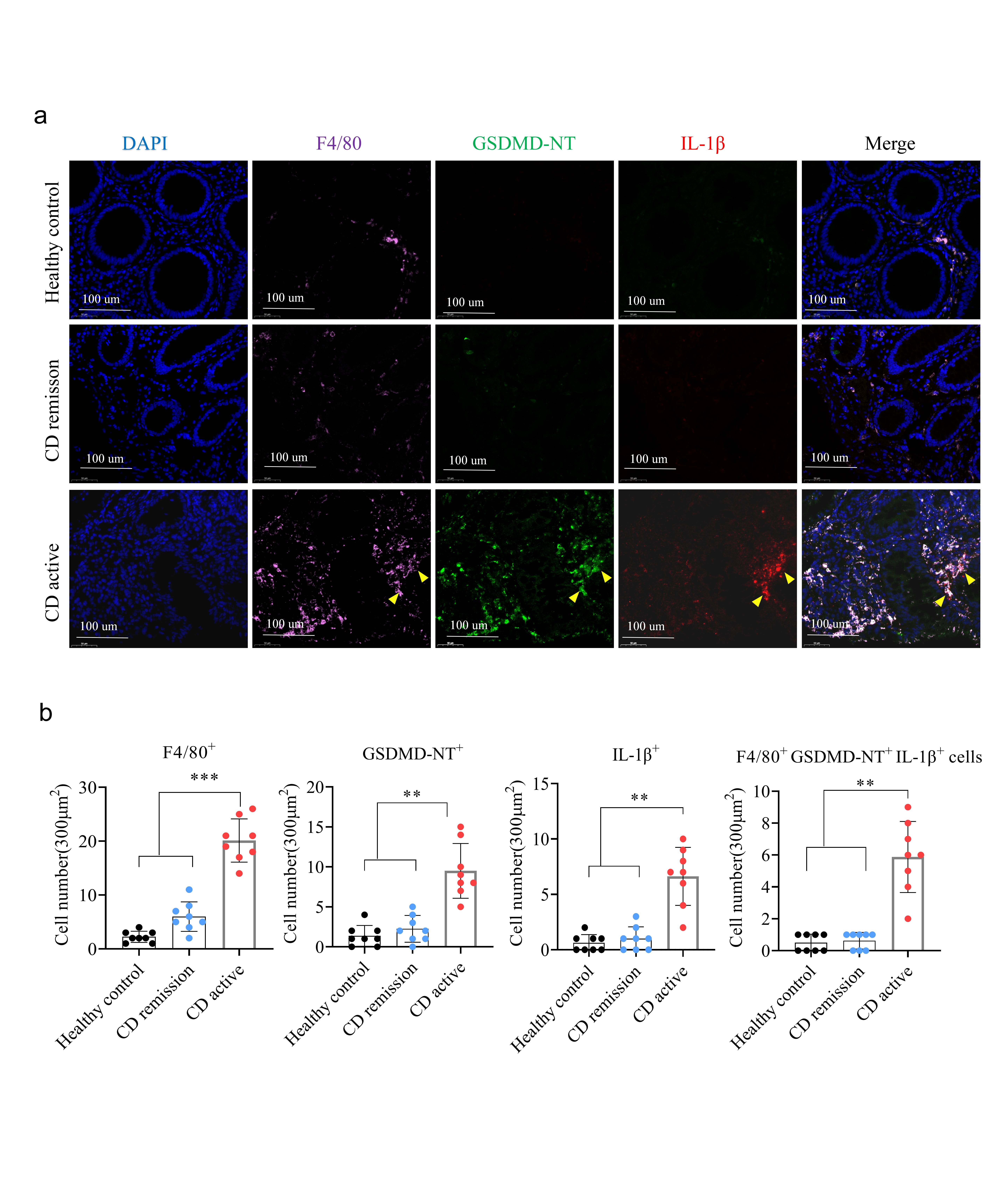


**Figure S8 GSDMD-medicated pyroptosis and IL-1β secretion are involved with CD development**

1. Representative immunofluorescence images of F4/80, GSDMD-NT and IL-1β immunostaining in colonic tissues (scale bars: 100 μm) from CD patients and healthy controls. The yellow arrow indicates the co-localized field of view.
2. The number of colonic F4/80^+^, GSDMD-NT^+^, and IL-1β^+^ immune cells per 300 μm^2^. Pyroptotic cells were co-immunostained with F4/80 (pink), GSDMD-NT(green) and IL-1β (red). F4/80^+^ GSDMD-NT^+^ IL-1β^+^ cells were counted under a microscope.

Data represent means ± SEM; **P < 0.01; ***P < 0.001; Unpaired Student’s *t*-test was performed for statistical analysis.


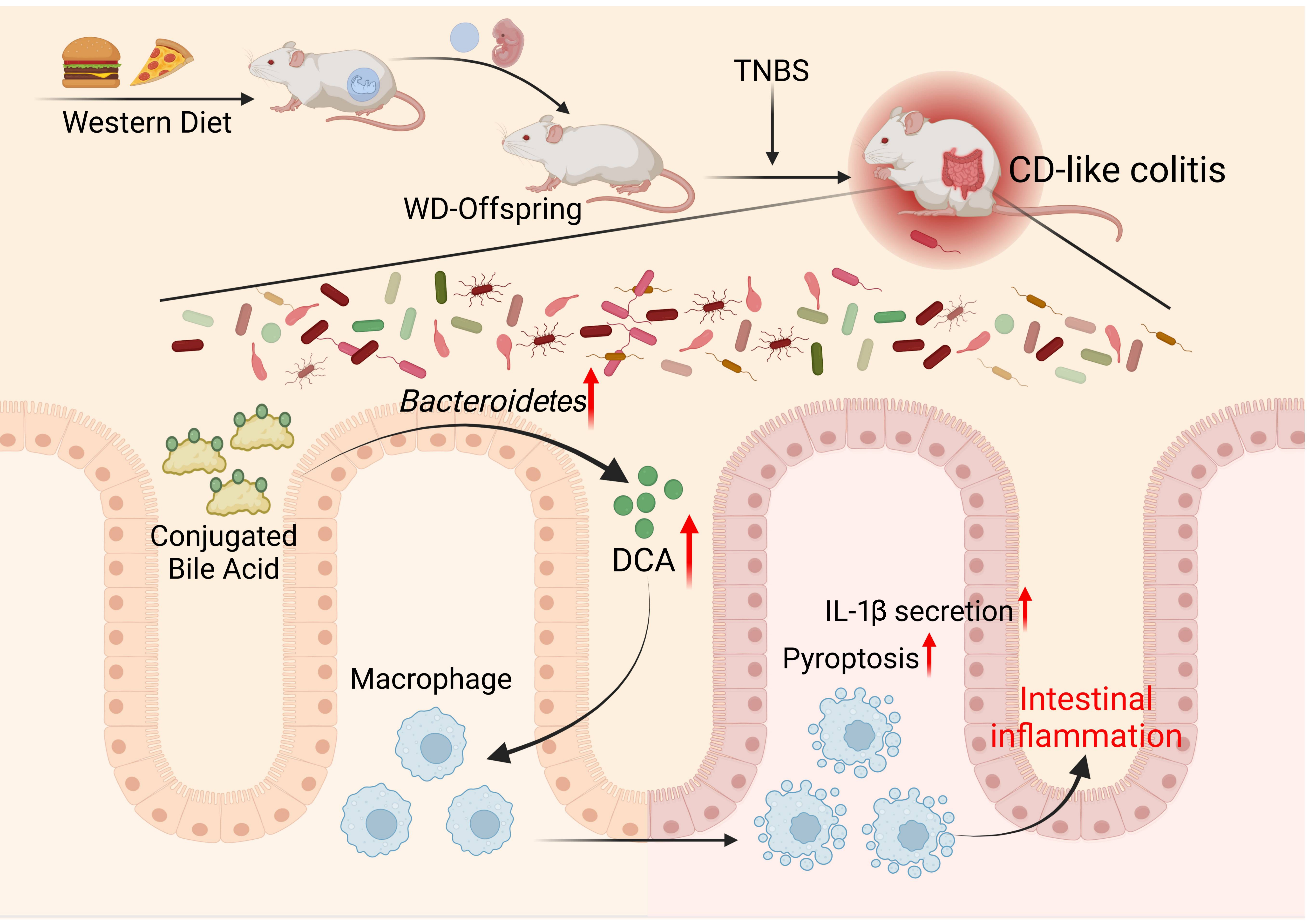


**Figure S9 Model showing how maternal Western diet influences offspring’s vulnerability to TNBS-induced CD-like colitis**

In this model, maternal exposure to a Western diet increased *Bacteroidetes*, which leads to higher DCA generation. In macrophages, elevated levels of DCA stimulate pyroptotic cell death and IL-1β release, worsening the development of TNBS-induced CD-like colitis in offspring.
